# Potential role of ochratoxin A in Parkinson’s disease: a systematic review of current evidence

**DOI:** 10.1007/s00204-025-03994-5

**Published:** 2025-03-06

**Authors:** M. Serrano-Civantos, E. Beraza, L. Álvarez-Erviti, A. López de Cerain, A. Vettorazzi

**Affiliations:** 1 Department of Pharmaceutical Sciences, MITOX Research Group, School of Pharmacy and Nutrition, University of Navarra, Pamplona, Spain; 2 Laboratory of Molecular Neurobiology, Center for Biomedical Research of La Rioja (CIBIR), Piqueras 98, 26006 Logroño, Spain

**Keywords:** Ochratoxin, Neurotoxicity, Adverse outcome pathway, Parkinson’s disease, Neurodegeneration

## Abstract

Ochratoxin A (OTA) is a mycotoxin produced by *Aspergillus* and *Penicillium* species that contaminates various food and feed products, presenting potential risks to human health. While OTA is well-known for its nephrotoxic effects, emerging evidence highlights its neurotoxic potential. Parkinson’s disease (PD) is a neurodegenerative disorder with both genetic and environmental aetiologies. Emerging lines of investigation have focused their research on the role of environmental toxins, including mycotoxins, in PD pathogenesis. However, the specific involvement of OTA in PD-related pathways still needs to be unravelled. This systematic review compiles and evaluates OTA neurotoxicity studies according to the adverse outcome pathway (AOP) for PD, established by the Organisation for Economic Cooperation and Development (OECD). The AOP framework outlines a series of key event (KEs) beginning with mitochondrial Complex I (CI) inhibition and progressing through mitochondrial dysfunction, impaired proteostasis, dopaminergic neuron degeneration, neuroinflammation, and resulting in parkinsonian motor deficits. In this systematic review, a comprehensive literature search was conducted in PubMed, to identify studies evaluating OTA neurotoxic effects. Using a search strategy of 19 terms and following a two-phased study selection, 30 relevant studies were retrieved, of which 16 dealt with in vitro adult neurotoxicity (ANT), 13 focused on in vivo ANT, and 1 gave both in vitro and in vivo approaches. Authors agree that in vitro and in vivo exposure to OTA causes mitochondrial dysfunction, impaired proteostasis, degeneration of dopaminergic (DA) neurons, and neuroinflammation. However, a notable absence of research remains on the molecular initiating event (MIE), binding to CI, and on KE1, inhibition of CI. This review identifies critical research gaps and highlights the need for further mechanistic studies on the impact of OTA on neurodegenerative pathways, particularly its binding and inhibition of CI, as well as mechanisms related to KE3: impaired proteostasis. Addressing these gaps may provide valuable insights into OTA neurotoxic potential and its relevance in PD-like neurodegeneration.

**Supplementary Information:**

The online version contains supplementary material available at 10.1007/s00204-025-03994-5.

## Introduction

Mycotoxins are secondary metabolites produced by filamentous fungi that contaminate fields and crops worldwide and exert toxic effects on plants, vertebrates and other animals (Bennett and Klich 2003). Among these, ochratoxin A (OTA) is one of the most relevant and detrimental mycotoxins. Indeed, in Europe, it is a regulated mycotoxin (Commission Regulation (EU) 2023/915 2023) and has been evaluated by the European Food Safety Authority (EFSA) two times (EFSA 2006, 2020). The first OTA producer identified was *Aspergillus ochraceus*, but it has also been isolated from other strains of *Aspergillus* and from *Penicillium*. OTA contaminates foodstuffs such as cereal products, raisins, beer, wine, coffee, pork blood products, and pork and chicken meat, as well as feedstuffs (Malir et al. 2016; Ben Miri et al. 2024). Its removal from these products is nearly impossible, due to its thermal stability, that allows this toxin to persist through most of the food processing procedures (Raters and Matissek 2008; Vidal et al. 2015). Furthermore, OTA has been detected in human blood, urine, milk, and kidneys at low concentrations, demonstrating that OTA contamination reaches up to humans (EFSA 2020).

The kidney is recognised as the principal target organ for OTA-induced toxicity (Khoi et al. 2021). This selectivity is largely attributed to the ability of OTA to accumulate in proximal tubule epithelial cells, inducing oxidative stress and DNA damage, promoting inflammation, and leading to cell death (Heussner & Bingle 2015). However, research over the years has also highlighted OTA’s neurotoxic potential, showing significant effects on brain structure and function. For instance, Miki et al., (1994) demonstrated that prenatal exposure to OTA in mice decreases cortical thickness through cell death. Additionally, it was observed that an intraperitoneal administration of OTA (0–6 mg/kg of b.w.) to mice, caused an acute depletion of striatal dopamine levels (Sava et al. 2006a). Moreover, a subcutaneous administration of OTA (4, 8, and 16 mg/kg of b.w.) by continuous infusion for 2 weeks, also resulted in a dose-dependent decrease in striatal dopamine in mice (Sava et al. 2006b). Later, Bhat et al. (2018) demonstrated that intraperitoneal administration of 3.5 mg OTA/kg b.w. for 3 days resulted in dopamine and other neurotransmitters levels and parkinsonian motor alterations in mice. More recent studies have explored the neurodegenerative effect of OTA, examining its potential link to neurodegenerative diseases with unclear etiology, such as Parkinson’s disease (PD). Izco et al. (2021) demonstrated, through in vivo studies with Balb/c mice, that 28 days OTA treatment p.o*.* (0.21 and 0.5 mg/kg of b.w.) induced motor alterations and dopaminergic (DA) dysfunction associated with the phosphorylation of α-synuclein (α-syn), at both intestinal and brain levels, detected six months after the end of OTA treatment. Additionally, a decrease in LAMP-2A (a protein involved in α-syn degradation process) protein levels, was reported in both midbrain and intestinal levels. Complementary in vitro studies indicated that subtoxic concentrations of OTA (100 and 200 nM) significantly elevated intracellular α-syn levels and its half-life while reducing LAMP-2A protein levels. These effects were observed in a neuroblastoma (SH-SY5Y) cell model, over-expressing wild-type (WT) human α-syn, and an intestinal (Caco-2) cell model, after 72 h OTA treatment. Altogether, these data point to a possible role of OTA in the etiology and progression of Parkinson’s disease, highlighting the importance of further examining its neurotoxic effects in relation to PD pathology development (Izco et al. 2021).

PD is a progressive neurodegenerative disorder characterised by the loss of dopaminergic neurons and the presence of Lewy Bodies (LBs) in the substantia nigra pars compacta (SNpc), which are primarily composed of α-syn aggregates (Elbaz et al. 2016). Mechanisms such as oxidative stress and disruptions in the autophagy-lysosome pathway (ALP) are thought to promote the aggregation of α-syn, potentially triggering neurodegeneration through processes like microglial activation and apoptosis (Marques and Outeiro 2012; Sarkar et al. 2016). These cellular changes underlie the hallmark motor symptoms of PD.

**Fig. 1:**
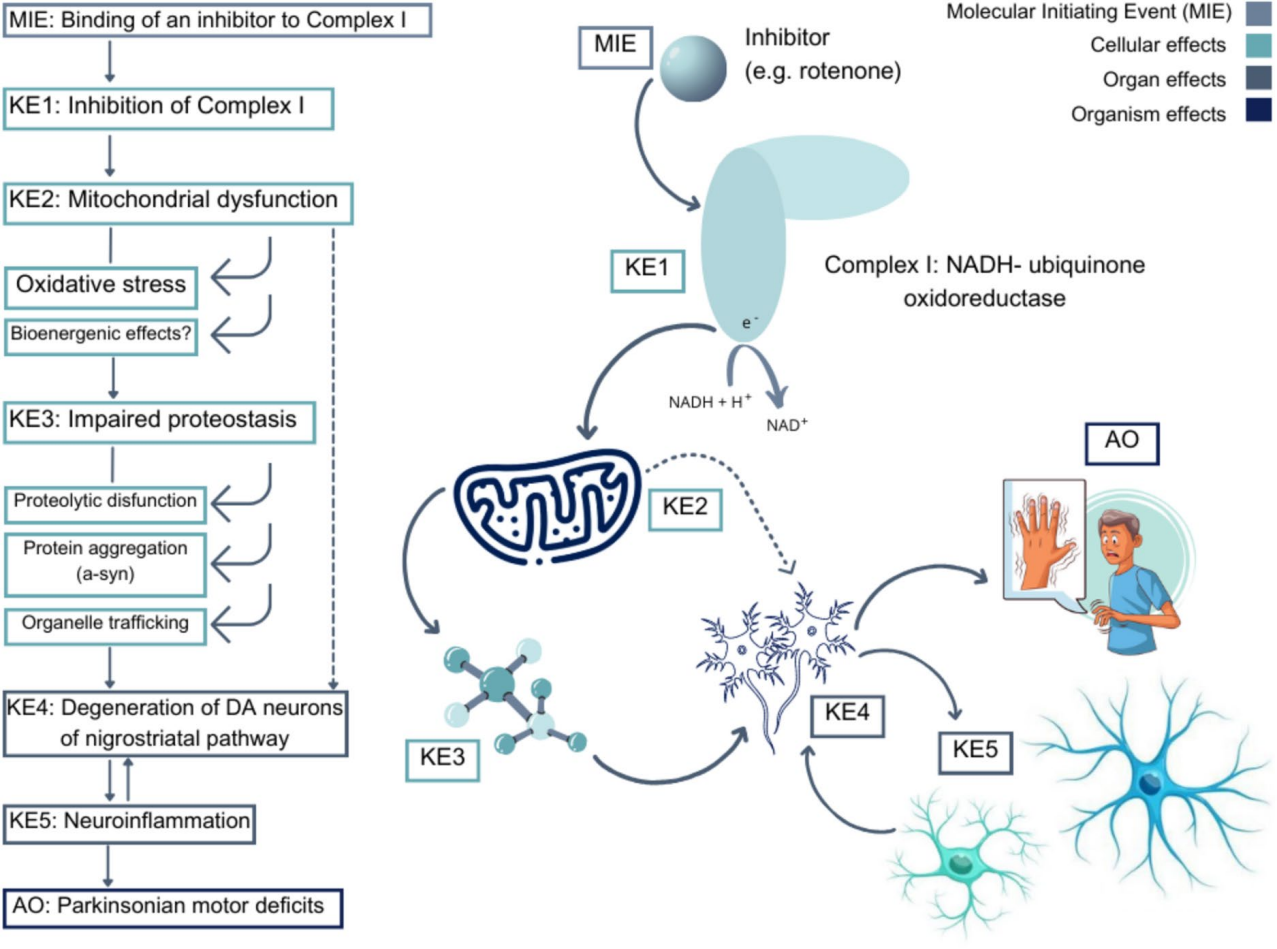
Parkinson’s disease adverse outcome pathway (AOP): inhibition of the mitochondrial complex I of nigrostriatal neurons leads to parkinsonian motor deficits. *α-syn* alpha synuclein, *AO* adverse outcome, *DA* dopaminergic, *KE *key event, *MIE* molecular initiating event. Information retrieved from https://aopwiki.org/aops/3

In 2012, the Organisation for Economic Cooperation and Development (OECD) launched a programme on the development of adverse outcome pathways (AOP). An AOP is a model that represents a sequence of biological events (molecular and cellular) linking a perturbation of a certain biological target by a stressor and its resultant adverse outcome(s). The OECD AOP related to PD describes the relation between the inhibition of the complex I (CI) of the mitochondrial respiratory chain of nigrostriatal neurons and the development of parkinsonian motor deficits (Bal-Price et al. 2018). It has been developed mainly based on effects observed after exposure to toxins such as rotenone or 1-methyl-4-phenyl-1,2,3,6-tetrahydropyridine (MPTP). The AOP starts with a molecular initiating event (MIE): the binding of an inhibitor to the complex I (NADH-ubiquinone oxidoreductase) (Fig. 1). The MIE is followed by 5 key events (KE): the inhibition of complex I (KE1); mitochondrial dysfunction (KE2): oxidative stress and bioenergetic effects; impaired proteostasis (KE3): i.e., proteolytic dysfunction, protein aggregation (α-syn) and organelle trafficking; degeneration of DA neurons of nigrostriatal pathway (KE4) and neuroinflammation (KE5). This OECD AOP is not a regulatory tool but could be applied to other chemicals with a similar structure to the stressors (i.e., rotenone or MPTP) or chemicals that can bind to complex I, to gain more mechanistic information.

OTA neurotoxicity mechanisms and their potential link to neurodegenerative diseases such as PD remain insufficiently understood. Given OTA large occurrence in food and feedstuff and its established impact on human health, investigating its neurotoxic and, more specifically, neurodegenerative potential is essential to further elucidate its role in the onset and progression of neurologic disorders. Thus, the aim of the present systematic review is to compile existing neurotoxicity studies on OTA, analysing these articles according to OECD AOP for PD. This approach will enable the identification of gaps in current knowledge regarding OTA’s neurotoxic mechanisms and highlight areas where further research is needed to clarify its possible involvement in PD-like neurodegeneration.

## Materials and methods

### Search strategy

**Fig. 2:**
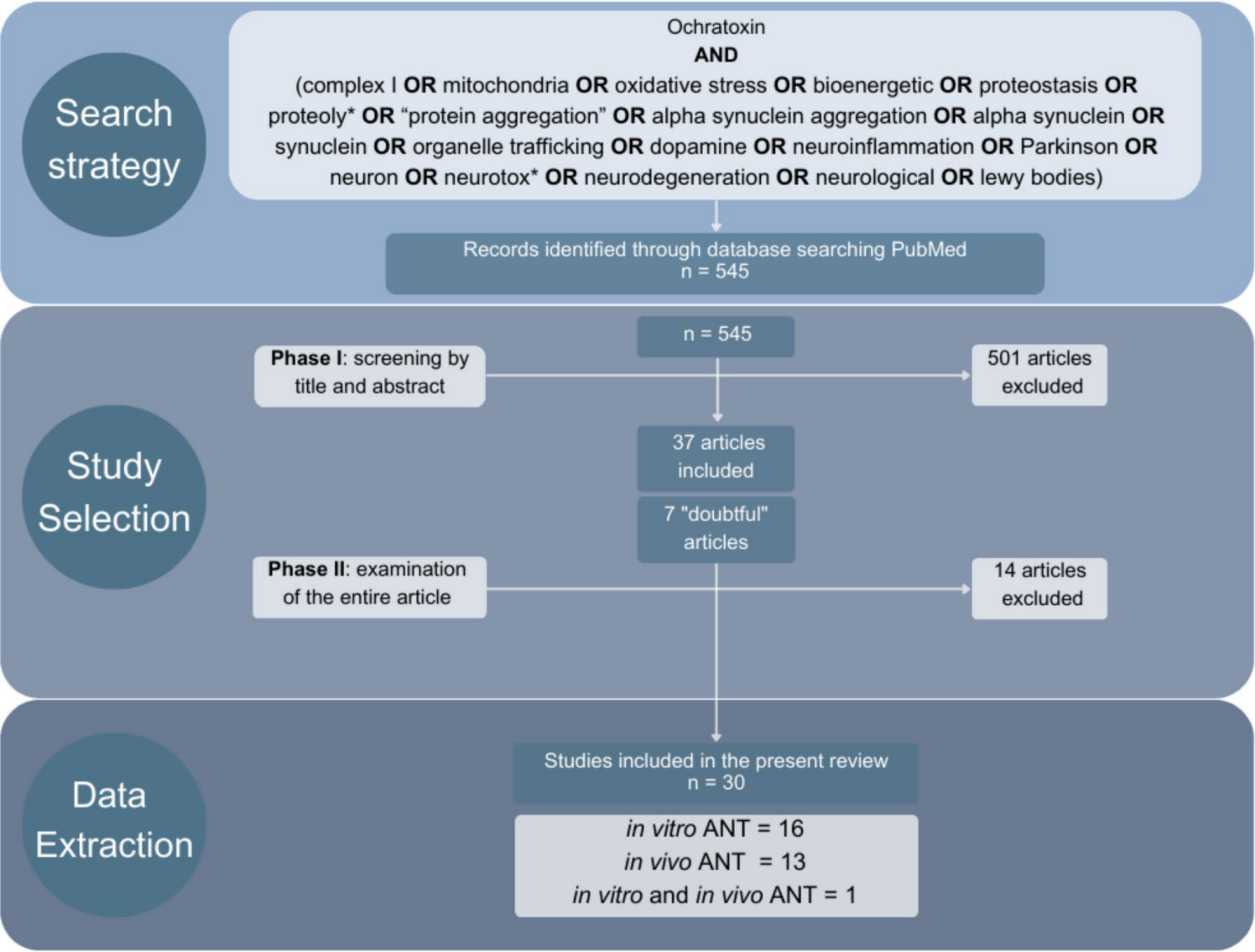
Search flowchart of the systematic literature search in PubMed. ANT: adult neurotoxicity

A systematic literature search in PubMed was performed to retrieve articles evaluating the potential neurotoxic effects of OTA and to analyse such articles according to the AOP of PD. The search was conducted on November 20, 2024, and no timeline filter was applied. The earliest retrieved article dated back to 1966. The search was carried out using the following keywords: “Ochratoxin AND (complex I OR mitochondria OR oxidative stress OR bioenergetic OR proteostasis OR proteoly* OR “protein aggregation” OR alpha synuclein aggregation OR alpha synuclein OR synuclein OR organelle trafficking OR dopamine OR neuroinflammation OR Parkinson OR neuron OR neurotox* OR neurodegeneration OR neurological OR lewy bodies)”. The search flowchart is shown in Fig. 2.

### Study selection

As PD is a long-term disease, the study selection focused only on adult neurotoxicity (ANT). The study selection was conducted in two phases: Phase I, which consisted of the screening of the articles by title and abstract; and Phase II, which consisted of the examination of the entire article (see Fig. 2).

#### Phase I

Titles and abstracts of all retrieved studies were screened and articles were selected using the following exclusion criteria:

1. Articles assessing the effects of a mycotoxin different than OTA.
2. Articles focused on a neurodegenerative disease different than PD.
3. Studies in which the main objective differs from neurotoxicity.
4. Studies dealing with developmental neurotoxicity.
5. Articles dealing with human epidemiological or clinical trials.
6. Reviews.

If the screening of the title and abstract did not provide enough information concerning the defined exclusion criteria to exclude a study, the article was labelled as “doubtful” and included in Phase II for further evaluation.

#### Phase II

Eligible articles or articles that were labelled as doubtful in Phase I, were retrieved and the entire texts were evaluated.

### Data extraction

Articles retrieved after Phase II were evaluated for data extraction. Data were collected as interpreted by the original publications; therefore, the interpretation of the authors was not altered by the authors of this review.

The information obtained from the selected articles was classified according to the assays that are described in the OECD PD AOP (Bal-Price et al. 2018) evaluating or measuring each KE (for a summary see Table 1). Accordingly, the KE of the AOP on which each study was focused was identified (e.g., mitochondrial dysfunction, impaired proteostasis…), as well as the assays performed to measure or detect such KE. Thus, tables relating each in vitro or in vivo assay with a specific KE were created, and all the articles were grouped chronologically by assay and KE (Tables 2 and 3, and Supplementary Tables 1, 2, 3, 4, 5, 6, 7, 8, and 9). Some information could not be associated with any AOP KE, in particular results from in vivo studies published between 1996 and 2010, and they were analysed separately.

**Table 1:** Classification of the assays to detect/measure the key events of Parkinson’s disease adverse outcome pathway*.*

Event	Assays that detect/measure the event
MIE: Binding of inhibitor to Complex I (CI)	Measurement of binding by quantitative autoradiography
	CI enzyme activity (colourimetric)
KE1: Inhibition of CI	Direct detection of CI activity:
	- Forward electron transfer - Reverse electron transfer - CI activity dipstick assay
	Indirect measurements of CI activity:
	- Oxygen consumption - Intracellular ATP levels
KE2: Mitochondrial dysfunction	Assays assessing a loss-of function:
	- Cellular oxygen consumption - Mitochondrial membrane potential (∆Ψm) - Enzymatic activity of the electron transport system - ATP content
	Assays assessing a gain-of function:
	- Mitochondrial permeability transition pore opening - mtDNA damage as a biomarker of mitochondrial disfunction - Generation of ROS and resultant oxidative stress: o ROS production by direct or indirect assays o Measurement of the cellular GSH status o Quantification of lipid peroxidation o Detection of superoxide production o Detection of H_2_0_2_ production
KE3: Impaired proteostasis	Evaluation of the UPS function:
	- General turnover assays - Proteasome activity assays - Detection of a-syn aggregates
	Evaluation of the ALP function:
	- Monitoring of autophagy-related molecules - Monitoring of the autophagic flux - Monitoring of the conversion of LC3-I to LC3-II
	Evaluation of intracellular transport of mitochondria and other organelles
KE4: Degeneration of DA neurons of the nigrostriatal pathway	Identification and quantification of biological markers by several histological and imaging techniques: - DA neurons in SNpc - DA terminals in the striatum - Dopamine content in the striatum - Lewy bodies
KE5: Neuroinflammation	Detection of microglial activation by quantification of cellular markers or released mediators by immunocytochemical methods, PCR or PET imaging
AO: Parkinsonian motor deficits	Detection of striatal dopamine
	Detection of DA neuron terminals in the striatum
	Behavioural tests: rodent models
	- Rotation/Rotarod/Hang test/Forepaw Stride length during walking/Grid test/Akinesia/Open field test/Pole test
	Non-invasive imaging of DA neuron terminals
	- Positron emission tomography (PET) - Single photon emission computed tomography (SPECT)

Information retrieved from https://aopwiki.org/aops/3

*ALP* autophagy-lysosome pathway, *CI *Complex I, *DA* dopaminergic, *LC3* microtubule-associated protein 1A/1B-Light Chain 3, *MIE* molecular initiating event, *KE* Key Event*, PCR* polymerase chain reaction, *PET* Positron Emission Tomography, *SPECT* Single photon emission computed tomography, *SNpc* Substantia nigra pars compacta, *UPS* ubiquitin proteasome system

**Table 2:** Summary of results obtained from reviewed articles assessing key events (KEs) of the Parkinson’s disease adverse outcome pathway (AOP) using in vitro techniques

Cell line	OTA concentration	Result	References
KE2: mitochondrial dysfunction			
*Intracellular ROS direct or indirect analysis*			
SK-N-MC cells	12.38, 24.76 and 49.53 μM	Dose-dependent increase of ROS	Baldi et al. 2004
SH-SH5Y and HT22 cells	100 μM	Increase of ROS in both cell lines	Yoon et al. 2009
Neuro-2a cells	100, 250 and 500 nM	Significant dose-dependent increase of ROS	Bhat et al. 2016
SH-SH5Y and HT22 cells	10 μM	Significant increase of ROS after 30 min. Maximal level of Fpg-sensitive sites detected after 1 h OTA treatment	Babayan et al. 2020
RGC-5 cells	248 and 496 nM	Dose-dependent increase of ROS	Fu et al. 2024
GHA cells	5–15 μM	Dose-dependent increase of ROS	Chu et al. 2024
*Quantification of lipid peroxidation*			
Primary rat neurons and astrocytes	10, 20, 25, 50, 75, 100, 150 μM	Dose-dependent MDA increase in both cell lines	Belmadani et al. 1999
Neuro-2a cells	100, 250 and 500 nM	Significant MDA increase at 250 and 500 nM OTA	Bhat et al. 2016
RGC-5 cells	248 and 496 nM	Dose-dependent increase of MDA levels	Fu et al. 2024
*Measurement of the cellular GSH status*			
RGC-5 cells	248 and 496 nM	Dose-dependent decrease of GST levels	Fu et al. 2024
GHA cells	5–15 μM	Dose-dependent reduction of GSH levels	Chu et al. 2024
*Detection of superoxide production*			
RGC-5 cells	248 and 496 nM	Dose-dependent decrease of SOD levels	Fu et al. 2024
*Measurement of Mitochondrial Membrane Potential (∆Ψm)*			
SH-SH5Y and primary rat neurons	0.1, 0.25, 1.0 and 2.5 μM	Dose-dependent loss of Δψm in both cell lines	Zhang et al. 2009
Neuro-2a cells	100, 250 and 500 nM	Dose-dependent loss of Δψm	Bhat et al. 2016
NHA-SV40LT cells	0.5, 1 and 2 μM	Significant loss of Δψm at 2 μM OTA	Park et al. 2019
RGC-5 cells	248 and 496 nM	Dose-dependent loss of Δψm	Fu et al. 2024
*Mitochondrial permeability transition pore opening (MPTPo) evaluation*			
NHA-SV40LT cells	0.5, 1 and 2 μM	Slight increase of intracellular Ca^++^ ions; Significant increase of mitochondrial Ca + + at 2 μM OTA	Park et al. 2019
KE3: impaired proteostasis			
*General turnover assays*			
WT α-syn SH-SH5Y cells	100 nM	OTA treatment significantly increased α-syn half-life (by 26%)	Izco et al. 2021
*Monitory of autophagy-related molecules*			
WT α-syn SH-SH5Y cells	100 and 200 nM	Significantly decrease of LAMP-2A protein and mRNA levels. No changes in hsc70 expression	Izco et al. 2021
KE5: neuroinflammation			
*Detection of astrocyte markers*			
Primary rat astrocytes	10 and 20 nM	Decrease of GFAP mRNA expression and GFAP staining. Dose-dependent increase of vimentin mRNA levels	Zurich et al. 2005
Primary rat astrocytes	10 μM	No significant effect over GFAP and GLT-1 total expression; 50% decrease in GFAP and GLT-1 cell surface expression	Razafimanjato et al. 2010
Primary rat astrocytes	10 nM	Decrease of MT1/MTII expression after 48 h of exposure and a decrease of GFAP mRNA levels after 24 h	von Tobel et al. 2014
*Glutamine synthetase (GS) assay*			
Primary rat astrocytes	10 and 20 nM	20 nM OTA significantly decreased GS mRNA levels and GS activity 48 h post-treatment	Zurich et al. 2005
Primary rat astrocytes	1, 10 and 100 μM	100 μM OTA significantly inhibited GS activity	Razafimanjato et al. 2010
*Detection of microglial activation*			
Oligodendrocytes and microglial cells	10 nM	Significant increase of IB4 positive cells. Increase of pro-inflammatory cytokines levels and decreased of anti-inflammatory cytokines Rise of Itgam and Cd86 levels and upregulated Mrc1 expression	von Tobel et al. 2014
BV-2 cells	50–2000 nM	A dose-dependent upregulation of IL-6, TNF-α, IL-1ß, and iNOS mRNA levels; as well as extracellular IL-6 and NO levels	Chansawhang et al. 2022
SH-SH5Y cells	3.1, 6.25, 12.5 μM	IL-6 and TNF-α expressions were slightly increased after 24 h OTA exposure and significantly increased after 48 h in all three doses	Penalva-Olcina et al. 2024

*α-syn* *α*-synuclein, *Cd86/B7-2* cluster of differentiation 86, *∆Ψm* mitochondrial membrane potential, *GFAP *glial fibrillary acidic protein, *GLT-1* glial glutamate transporter type 1, *GS* glutamine synthetase, *GSH* glutathione, *GST* Glutathione-S-transferase, *hsc70 *heat shock cognate protein 70, *IL* interleukin, *IB4* isolectin B4, *iNOS* inducible nitric oxide synthase, *Itgam/Cd11b* integrin alpha M, *LAMP-2A* lysosome-associated membrane protein 2A, *MDA* malondialdehyde, *MPTPo* mitochondrial permeability transition pore opening, *Mrc1/Cd206* mannose receptor C type 1, *MTI/II* melatonin receptor type 1 and 2, *NO* nitric oxide, *OTA* Ochratoxin A, *ROS* Reactive Oxygen Species, *SOD* superoxide dismutase, *TNF* tumour necrosis factor.

**Table 3:** Summary of results obtained from reviewed articles assessing key events (KEs) of the Parkinson’s disease adverse outcome pathway (AOP) using in vivo techniques

Experimental system	Route	Dose (mg/kg b.w.)	Results	References
KE2: mitochondrial dysfunction				
*Intracellular ROS indirect analysis*				
Swiss ICR mice	i.p	0–6	Increased oxidative DNA damage in all brain regions at all time points. Inhibition of OGG1 activity in all brain regions	Sava et al. 2006a
*Quantification of lipid peroxidation*				
Swiss ICR mice	i.p	0–6	MDA levels increased in a time-dependent way in all brain regions after OTA administration	Sava et al. 2006a
Balb/C albino mice	i.p	3.5	MDA levels were significantly increased in brain tissue after OTA administration	Bhat et al. 2018
Albino Wistar rats	p.o	10	MDA levels were significantly increased (50%) in brain tissue after OTA administration	Nogaim et al. 2020
WT zebrafish	i.p	1.38, 2.77, 5.53 mg/kg b.w	No changes were observed in TBARS levels after OTA administration compared to controls	Valadas et al. 2023
*Measurement of the cellular GSH status*				
Balb/C albino mice	i.p	3.5 mg/kg b.w	GSH status was significantly reduced after OTA administration	Bhat et al. 2018
Albino Wistar rats	p.o	10 mg/kg b.w	GSH levels were significantly reduced (27%) in brain tissue after OTA administration	Nogaim et al. 2020
*Detection of superoxide production*				
Swiss ICR mice	i.p	0–6 mg/kg b.w	Upregulation of SOD activity in all brain regions (33%), after OTA administration	Sava et al. 2006a
Balb/C albino mice	i.p	3.5 mg/kg b.w	SOD levels were significantly decreased in OTA-treated group compared to control groups	Bhat et al. 2018
Albino Wistar rats	p.o	10 mg/kg b.w	SOD activity was significantly decreased in brain tissue (33%) after OTA treatment	Nogaim et al. 2020
KE3: impaired proteostasis				
*Monitoring of autophagy-related molecules*				
Balb/C albino mice	p.o	0.21, 0–5 mg/kg b.w	Dose-dependent decrease in LAMP-2A (20% at 0.21 mg/kg b.w. and 50% at 0.5 mg/kg b.w.) in midbrain, while no changes were observed in hsc70 protein levels	Izco et al. 2021
KE4: degeneration of dopaminergic neurons of the nigrostriatal pathway				
*Dopaminergic neurons in the striatum/SNpc*				
Balb/C albino mice	p.o	0.21, 0–5 mg/kg b.w	Significant decrease of TH staining (loss of DA striatal innervation). Significant decrease (26%) in the number of TH + DA neurons in the midbrain	Izco et al. 2021
*Dopamine content in the striatum/SNpc*				
Swiss ICR mice	i.p	0–6 mg/kg b.w	Dose-dependent decrease of dopamine striatal content (50%). ED50 of 3.2 mg/kg b.w	Sava et al. 2006a
Swiss ICR mice	s.c	4, 8, 16 mg/kg b.w	Dopamine content decrease by 24% in caudate/putamen, after 2 cumulative dose of 8 mg/kg b.w	Sava et al. 2006b
Balb/C albino mice	i.p	3.5 mg/kg b.w	Dopamine content decrease in hippocampus, striatum and whole brain tissue compared to control	Bhat et al. 2018

Experimental system	RoA	OTA dose	Result	References
KE4: degeneration of dopaminergic neurons of the nigrostriatal pathway				
*Detection of Lewy Bodies (α-syn)*				
Balb/C albino mice	p.o	0.21, 0–5 mg/kg b.w	p-syn aggregates in SNpc were detected (0.21 mg OTA/kg b.w.: 1.5 aggregates/section; 0.5 mg OTA/kg b.w.: 2.5 aggregates/ section)	Izco et al. 2021
KE5: neuroinflammation				
*Detection of astrocyte markers*				
C57BL/6 mice	i.p	3.5 mg/kg b.w	A significant dose-dependent decrease in GFAP cell expression was observed (60% after six cumulative doses)	Mateo et al. 2022
Adverse Outcome: parkinsonian motor deficits				
*Behavioural tests*				
Adult sea bass	p.o	0.05, 0.1, 0.15, 0.2, 0.25, 0.3, 0.35, 0.4 mg/kg b.w	Behavioural changes: sluggish movement, loss of equilibrium, rapid operculum movement as respiratory manifestations. Before death, muscular seizures occurred	El-Sayed et al. 2009
Balb/C albino mice	i.p	3.5 mg/kg b.w	Gait analysis: reduced mean stride length measurements of forelimb and hindlimb. Spontaneous activity: lower activity indicated by forelimb and hindlimb steps, rears in cylinder and grooming time. Adhesive removal: significant longer time taken for making contact and removing the stimulus. Parallel bars: significant longer time taken to orient themselves and to walk to one end of the pole, showing a decreased motor coordination. Pole test: significantly affected extrapyramidal motor dexterity	Bhat et al. 2018
Balb/C albino mice	p.o	0.21, 0–5 mg/kg b.w	Significant decrease in motor performance was detected by the wire hang test and the negative geotaxis test in OTA-treated mice, 31 weeks AET	Izco et al. 2021
WT zebrafish	i.p	1.38, 2.77, 5.53 mg/kg b.w	Open tank test: significant decrease in distance, absolute turn angle and mean speed. Increase in freezing time, indicating locomotor impairment Social interaction test: no altered social behaviour in any of the analysed parameters (distance, crossings, and interaction)	Valadas et al. 2023

*α-syn* *α*-synuclein, *Cd86/B7-2* cluster of differentiation 86, *∆Ψm *mitochondrial membrane potential, *GFAP* glial fibrillary acidic protein, *GLT-1* glial glutamate transporter type 1, *GS* glutamine synthetase, *GSH* glutathione, *GST* Glutathione-S-transferase*, hsc70* heat shock cognate protein 70, *IL* interleukin, *IB4* isolectin B4, *iNOS* inducible nitric oxide synthase, *i.p.* intraperitoneal, *Itgam/Cd11b* integrin alpha M, *LAMP-2A* lysosome-associated membrane protein 2A, *MDA* malondialdehyde, *MPTPo* mitochondrial permeability transition pore opening, *Mrc1/Cd206* mannose receptor C type 1, *MTI/II* melatonin receptor type 1 and 2, *NO *nitric oxide, *OTA* Ochratoxin A, *p.o* *per os* (oral), *RoA* route of administration, *ROS* Reactive Oxygen Species, *s.c. *subcutaneous, *SOD* superoxide dismutase, *TNF* tumour necrosis factor

## Results

After applying the search strategy, a total of 545 articles were retrieved. In Phase I of the study selection, 501 articles were rejected following the exclusion criteria, 37 articles were included, and 7 articles were identified as “doubtful”. All 7 “doubtful” studies were excluded in Phase II, as well as 7 of the 37 articles included in Phase I. Notably, some of the articles met multiple exclusion criteria. In total, 515 articles were excluded, the breakdown of exclusion as follows: 449 articles were excluded because the main objective differed from neurotoxicity, 66 articles were discarded because they were reviews, of which 41 also met other exclusion criteria. Other 3 articles were excluded for assessing the effects of a mycotoxin different from OTA, while 4 articles were excluded for dealing with human epidemiological or clinical trials and other 2 articles for being focused on a neurodegenerative disease different from PD. Finally, 32 studies were discarded for dealing with developmental neurotoxicity.

At the end of Phase II, a total of 30 articles were retrieved from the systematic search. More specifically, the search resulted in 16 studies assessing neurotoxicity using in vitro techniques and 13 using in vivo techniques (Fig. 2). 1 article used both in vitro and in vivo methods.

As a first step, an analysis of the different KE of the PD AOP addressed in the retrieved articles was performed. Each one of the key events can be detected or measured by one or more assays (Table 1). Some of these assays can be applied only in vivo or ex vivo, only in vitro, or in both experimental systems.

### Neurotoxicity by in vitro techniques

Among the 16 articles dealing with ANT by in vitro techniques, 8 studies were focused on mitochondrial dysfunction (KE2), 1 article assessed impaired proteostasis (KE3), and 5 studies evaluated neuroinflammation (KE5). Assays related with KE1 (inhibition of CI) and KE4 (degeneration of DA neurons of the nigrostriatal pathway) were not found in the systematic search for OTA. The results obtained with in vitro assays related to each key events (KE2, KE3 and KE5) are detailed in Supplementary Tables 1, 2, and 3, respectively, and summarised in Table 2.

#### KE2: mitochondrial dysfunction

Mitochondrial dysfunction can be assessed through several assays that detect ROS production and its consequent oxidative stress and lipid peroxidation; as well as through the measurement of mitochondrial impairment markers, such ∆Ψm or the opening of the mitochondrial permeability transition pore (Supplementary Table 1). Table 2 presents all the information related to mitochondrial dysfunction from OTA in vitro studies, which are detailed further below.

##### Intracellular ROS production

For a direct analysis of intracellular ROS generation, methods based on oxidation-sensitive dyes were used: dichlorofluorescin diacetate (DCFH-DA) and dihydroethidium (DHE) staining assays. A significant increase in ROS was always obtained after treatment of human neuroblastoma or mouse neural cells with different OTA concentrations (12.38, 24.76 and 49.53 μM; 100 μM; 100, 250, and 500 nM) at exposure times from 30 min to 1 h, 24 h or 48 h (Baldi et al. 2004; Yoon et al. 2009; Bhat et al. 2016, respectively).

Babayan et al. (2020), using the DHE assay oxidation-sensitive dye technique that detects superoxide formation (O_2_^−^), also obtained a significant increase in ROS production after 30 min exposure, remaining during 6 h of incubation in human SH-SHSY cells, but gradually decreased in mice HT22 cells. They also carried out an indirect analysis of intracellular ROS production by means of an Fpg-comet assay, which detects DNA damage caused by oxidative stress. The highest level of Fpg-sensitive sites was detected after 1 h of treatment and a significant decrease was noticed during the 72 h of incubation in SH-SY5Y cell line and after 24 h of incubation in HT22 cells. These results implied a greater repair capacity of oxidative DNA lesions in the mouse cell line than in the human cell line. Moreover, Fu et al. (2024) also detected a dose-dependent increase in ROS production when exposing retinal ganglion cells (RCG-5) to 248 and 496 nM OTA for 3 days, and Chu et al. (2024) demonstrated that OTA also increases ROS production in human astrocytes (GHA cells) in a dose-dependent manner (5–15 μM OTA for 24 h).

Thus, OTA may be diminishing cell viability and inducing DNA damage via ROS generation. Furthermore, the rise of ROS levels can result in other negative consequences for the cell: free radicals can cause lipid peroxidation, which leads to a loss of mitochondrial membrane potential, which is associated with apoptotic processes.

##### Lipid peroxidation quantification

During lipid peroxidation, malondialdehyde (MDA) is formed as an end product, being then an adequate indicator of tissue damage caused by ROS. MDA can be detected through a method based on its reaction with thiobarbituric acid, forming a fluorescent complex. This method was used by Belmadani et al. (1999) (10, 20, 25, 50, 75, 100, 150 μM OTA) and Bhat et al. (2016) (100, 250, and 500 nM OTA), as well as by Fu et al. (2024) (248 and 496 nM OTA) to measure lipid peroxidation caused by OTA in rat primary neurons and astrocytes, Neuro-2a cells and RCG-5 cells respectively (Table 2 and Supplementary Table 1). A dose-dependent increase in MDA content was detected in all cell types.

##### Measurement of the cellular glutathione (GSH) status

GSH (glutathione reduced form), is one of the gamma-glutamyl-peptides and acts as an antioxidant that protects cells from oxidative stress by neutralising ROS. The enzyme Glutathione-S-Transferase (GST) uses GSH to conjugate and detoxify harmful compounds, enhancing their solubility for excretion. Together, they play a vital role in maintaining cellular redox balance and protecting against oxidative damage. Fu et al. (2024) measured GST levels as an indicator of oxidative stress in retinal RCG-5 cells, detecting a significant decrease in such levels after exposure to 248 and 496 nM OTA. On the other hand, Chu et al. (2024) observed a significant reduction of GSH levels in OTA-treated astrocytes (5–15 μM OTA), indicated by a decrease in fluorescence intensity. Given that both GST and GSH levels were decreased in response to OTA exposure, its role in impairing antioxidant defences and exacerbating oxidative stress is highlighted.

##### Detection of superoxide (O_2_^−^) production

Superoxide dismutases (SOD) are enzymes that catalyse the dismutation of superoxide into O_2_ and H_2_O_2_. By measuring the activity of these enzymes, the production of O_2_^−^ can be specifically detected. Fu et al. (2024) carried out a SOD assay, detecting a decrease in its levels in RGC-5 cells after 24 h of OTA exposure (248 and 496 nM OTA). This further indicates that OTA impairs the cellular antioxidant defence system.

##### Mitochondrial permeability transition pore opening (MPTPo)

The MPTPo can be evaluated by determining the Ca^++^ influx, using Fluo-4 dye to detect cytosolic Ca^++^ and Rho-2 dye to detect mitochondrial Ca^++^ (Park et al. 2019). Cytosolic Ca^++^ was slightly increased in normal human astrocytes (NHA-SV40LT) after treatment with 0.5, 1, and 2 μM OTA, while mitochondrial Ca^++^ levels were highly increased (Table 2 and Supplementary Table 1). An elevated intracellular Ca^++^ is one of the characteristics of reactive astrocytes and, as aforementioned, can trigger loss of ∆Ψm and apoptotic processes, as confirmed by the latter author and colleagues.

##### Mitochondrial membrane potential (∆Ψm)

The ∆Ψm can be disrupted during mitochondrial dysfunction, due to an increased Ca^++^ influx. To measure ∆Ψm as an indicative of mitochondrial dysfunction, JC-1 staining was used by several authors after OTA treatment (Table 2 and Supplementary Table 1). Through this method, a loss of ∆Ψm was observed when treating human neuroblastoma cells (SH-SY5Y) and primary rat neurons with 0.1, 0.25, 1.0 and 2.5 μM OTA (Zhang et al. 2009), human astrocytes (NHA-SV40LT) with 0.5, 1 and 2 μM OTA (Park et al. 2019) and retinal RCG-5 cells with 248 and 496 nM OTA (Fu et al. 2024). Another cationic dye, rhodamine 123, can be employed to analyse the ∆Ψm loss. Through this technique, a loss of ∆Ψm was detected in mouse Neuro-2a cells after exposure to 100, 250, and 500 nM for 24 h (Bhat et al. 2016).

A loss of ∆Ψm can lead to the release of cytochrome c, which in turn can activate other apoptotic factors. Thus, after reporting a loss of ∆Ψm when treating two neural cell lines with OTA, Zhang et al. (2009), proceeded to determine caspase-3, -8 and -9 protein levels through Western Blot (WB). An upregulation in caspase-3 and -9 protein expression was detected in SH-SY5Y cells and primary rat neurons treated with OTA. Interestingly, to assess whether OTA induced apoptosis via the mitochondria-caspase pathway, the authors treated both cell lines with two caspases inhibitors, which indeed were able to prevent OTA-induced cell death. This activation of caspase 3 after OTA treatment was confirmed by Bhat et al. (2016). In contrast, Yoon et al. (2009) only detected an activation of caspase-3 in OTA-treated HT22 cells, while non-activated caspase-3 was observed in SH-SY cells after OTA (10 or 100 μM, 24 h) exposure.

Also, Park et al. 2019 reported an upregulation of mitochondria-dependent apoptotic genes BAX and TP53 mRNA expression after exposing NHA-SV40LT cells to OTA (0.5, 1 or 2 μM) for 48 h. Yoon et al. (2009) studied through WB the effects of OTA (10, 100 μM) treatment over the levels of phosphorylated p53 on serine 15, being this residue an apoptosis-related site. No changes in OTA-treated SH-SY5Y cells were observed, while a dose-dependent increase on phosphorylated p53 (Ser^15^) was detected in HT22 cells.

In contrast, Sharma et al. (2023) reported no significant changes in BAX and p53 mRNA expression after treating SH-SY5Y cells with 2 pM OTA, while BDNF mRNA levels were significantly reduced after 11 days of OTA exposure. These authors carried out another experiment in similar conditions, but this time exposing SH-SY5Y cells to a higher OTA concentration (1 μM). After 1 and 2 days of treatment, BAX, P53, MAPT mRNA levels were decreased, while TPPP and DDNF expressions were decreased at day 1 but increased on day 2. Notably, these findings reported by Sharma et al. (2023) could not be related to any KE of the AOP.

Taken together, these data suggest that OTA induces the depolarisation of the mitochondrial membrane, triggering the activation of caspases-9 and -3, leading to mitochondria-dependent apoptosis.

#### KE3: impaired proteostasis

To assess an impairment in proteostasis, the ALP function can be studied, through the monitoring of ALP-related molecules. General turnover assays can also be performed. Only one article has been found in the search studying the in vitro effects of OTA upon impaired proteostasis (Table 2 and Supplementary Table 2).

In 2021, Izco et al. assessed the half-life of α-syn in a neuroblastoma cell line, genetically modified to overexpress wild-type full-length human alpha synuclein (WT α-syn SH-SY5Y), using a standard cycloheximide method. The cycloheximide is used to inhibit protein synthesis; α-syn was detected by WB. OTA (100 nM) significantly increased α-syn half-life by 26%. The decrease in α-syn turnover was related with a downregulation of LAMP-2A mRNA expression after 200 nM OTA exposure and a decrease in LAMP-2A protein levels after 100 and 200 nM OTA treatment. Levels of hsc70 remained unchanged.

#### KE5: neuroinflammation

Neuroinflammation is the activation of glial cells. This activation can be measured by quantifying specific cellular markers, or by quantifying cytokines released to the medium. Supplementary Table 3 presents all the information related to neuroinflammation from OTA in vitro studies, which is detailed further below and summarised in Table 2.

##### Detection of astrocyte markers

The most frequently used astrocyte marker is the glial fibrillary acidic protein (GFAP), although other markers such as vimentin, the glial glutamate transporter type 1 (GLT-1) or the melatonin receptors 1 and 2 (MTI/II) are used for staining of astrocytes. The most common techniques for the detection of these markers are immunostaining methods and RT-qPCR for the quantification of mRNA levels. Zurich et al. (2005) applied both techniques for the detection of GFAP and vimentin in rat primary astrocytes treated with 10 and 20 nM OTA for 24 h and 48 h. GFAP mRNA expression was significantly reduced by both OTA doses as well as the GFAP staining intensity. Regarding vimentin, OTA upregulated its mRNA levels in a dose-dependent way, while the staining intensity presented no changes. These results suggest that OTA induces alterations in the astrocytic cytoskeleton, affecting the GFAP/vimentin proportions. Other authors (Razafimanjato et al. 2010) also measured GFAP and GLP-1 total expression through immunostaining (cell-ELISA assay), detecting no significant changes. However, they performed a biotinylation technique to measure the cell surface expression of both markers and they observed that after 72 h of 10 μM OTA exposure, the cell surface expression of GFAP and GLP-1 decreased by 50%. The downregulation of GFAP expression by OTA in primary rat astrocytes was confirmed by Von Tobel et al. (2014) through RT-qPCR and biotinylation techniques. GFAP mRNA expression was significantly diminished after exposure to 10 nM OTA for 24 h. Melatonin receptor type 1 and 2 (MTI and MTII) expression was also significantly decreased by 10 nM OTA, suggesting that the mycotoxin not only affects astrocytic cytoskeleton as previously demonstrated (Zurich et al. 2005), but also induces alterations in astrocyte function.

Although glutamine synthetase assays are not included by the AOP as techniques to measure neuroinflammation, some authors considered them to be useful neuroinflammation indicators. Astrocytes absorb glutamate from the extracellular space and transform it into glutamine by the action of an enzyme called glutamine synthetase (GS). Glutamine is then released and reabsorbed by neurons that will transform it back into glutamate. Alterations in this glutamate-glutamine cycle is associated with neuroinflammation and neurological diseases such as PD (Zurich et al. 2005). To investigate this, this author and colleagues determined GS activity through a colourimetric assay, as well as its mRNA expression by means of an RT-qPCR technique. They observed that 20 nM OTA significantly decreased both GS mRNA expression and activity after 48 h of treatment. The GS activity inhibition was confirmed by (Razafimanjato et al. 2010) but only at the OTA concentration of 100 μM, which was found to be high cytotoxic for this cell line (primary rat astrocytes).

##### Detection of microglial activation

Neuroinflammation is characterised by the neurodegenerative M1 microglial activation state. Therefore, the measurement of markers of M1 microglial phenotype is indicative of a neuroinflammatory response. It has been demonstrated that repeated exposure to low concentrations of OTA (10 nM) induces the activation of microglial cells, triggering a neuroinflammatory response that ultimately results in neurodegeneration (Von Tobel et al. 2014). Several markers can be measured to assess microglial activation. The latter author and colleagues detected isolectin B4 (IB4) through an immunolabelling technique to evaluate changes in cell number and morphology; M1 (IL-4, IL-6, IL-1ß, TNF-α, Itgam/Cd11b and Cd86/B7-2) and M2 (Arg1 and Mrc1/Cd206) markers were determined through RT-qPCR method. After a 10-day exposure to 10 nM OTA, a significant increase in IB4-positive cells and in the expression of pro-inflammatory cytokines (IL-6, IL-1ß, TNF-α), and a decrease in the expression of anti-inflammatory cytokines (IL-4) were observed. Itgam and Cd86 expression was also upregulated by OTA after 48 h and 10 days exposure. On the other hand, no changes in Arg1 expression were detected, whereas Mrc1 expression was significantly increased at both time points. All these together indicate that OTA favours M1 microglial activation state at an early phase (48 h) but that the phenotype needs a longer period (10 days) to reach full activation. Also, the OTA-induced increase of Mrc1 levels suggests that a small portion of microglia alternatively acquires M2 phenotype.

This increase in pro-inflammatory cytokines was also observed when exposing microglial cells to OTA for shorter periods of time but at higher concentrations (Chansawhang et al. 2022; Penalva-Olcina et al. 2024). The mRNA expression of IL-6, IL-1ß, TNF-α, and iNOS was upregulated in a dose-dependent manner after 24 h OTA exposure (50–2,000 nM) in BV-2 cells (Chansawhang et al. 2022). The amount of nitric oxide (NO) and IL-6 released into the extracellular matrix was also determined and a dose-dependent (50–2000 nM OTA) increase was observed (Chansawhang et al. 2022). These authors hypothesised that OTA may trigger microglia activation via MAPKs pathway. Thus, microglial cells were pre-treated with different MAPKs molecules (ERK, p38 MAPK, and JNK) inhibitors for 1 h, and the co-treated with 50, 250, and 500 nM OTA for 24 h. Cells treated with ERK and p38 MAPK inhibitors significantly decreased IL-6, IL-1ß, TNF-α, and iNOS mRNA expression induced by OTA. On the other hand, through WB techniques, it was observed that OTA induced a significant increase in ERK and p38 MAPK phosphorylation levels. All these together indicated that OTA activated microglia through the activation of ERK and p38 MAPK pathways.

Penalva-Olcina et al. (2024) demonstrated that, when exposing neuroblastoma cells (SH-SY5Y) to micromolar doses of OTA (3.1, 6.25, 12.5 μM) for 24 h and 48 h, an increase in IL-6 and TNF-α production was observed, correlating with an increase of the SubG0 phase of the cell cycle, being this an indicator of cell death via the apoptosis pathway. Thus, these results point to the inflammatory effect of OTA over SH-SY5Y, which results in neurotoxicity.

### Neurotoxicity by in vivo techniques

Among the 14 articles dealing with ANT by in vivo techniques, 1 study was focused on mitochondrial dysfunction (KE2), 1 study was focused on the degeneration of DA neurons on the nigrostriatal pathway (KE4), 1 article studied neuroinflammation (KE5) and 1 study evaluated the adverse outcome. Four articles studied more than one KE. The results obtained with in vivo assays related to the KE2, KE3, KE4, and KE5 and the AO are detailed in Supplementary Tables 4, 5, 6, 7 and 8 respectively, and summarised in Table 3. No assays related to KE1 (Inhibition of CI) were found in the systematic search for OTA. Six studies were focused on endpoints which were not related to the AOP of PD (Supplementary Table 9).

#### KE2: mitochondrial dysfunction

Mitochondrial dysfunction can be assessed by several assays that measure ROS generation and the resultant oxidative stress and lipid peroxidation. Supplementary Table 4 presents all the information related to mitochondrial dysfunction from OTA in vivo studies, which is detailed further below and summarised in Table 3.

##### Intracellular ROS indirect analysis

The intracellular production of ROS was indirectly measured by assessing the ability to repair DNA oxidative damage. This was carried out by Sava et al. (2006a) by measuring the activity of the enzyme oxyguanosine glycosylase (OGG1). The activity of this enzyme was observed to be significantly decreased in all brain structures (cerebellum, pons/medulla, midbrain, hippocampus, caudate/putamen, and cortex) of Swiss ICR mice, 6 h after intraperitoneal (i.p.) exposure to 3.5 mg OTA/kg b.w., followed by a gradual return to control levels by 72 h. Interestingly, these authors also studied the DNA damage in brain tissue, using the comet assay in its standard version. It was observed that OTA exposure produced an increase in DNA damage across every brain region, which results in a significant inverse correlation between OGG1 activity and the levels of DNA damage at all timepoints, except at 72 h.

##### Lipid peroxidation quantification

As mentioned above, MDA is formed as a result of lipid peroxidation and therefore can be used as an indicator of such event. The most common technique to detect MDA levels is the thiobarbituric acid reactive substances (TBARS) assay, of which there can be found different modifications, but is basically based on the reaction between MDA and trichloroacetic acid (TCA) and/or thiobarbituric acid (TBA). Using this technique, Sava et al. (2006a), observed that i.p administration of 3.5 mg OTA/kg. Significantly increased TBARS levels in a time-dependent manner (6 h, 24 h, and 72 h), in all brain structures. Similarly, Bhat et al. (2018) also demonstrated that 3.5 mg OTA/kg i.p. increased TBARS levels in brain homogenates of mice treated with OTA for 3 consecutive days.

On the other hand, Valadas et al. (2023) used the same method to show that i.p administration of 1.38, 2.77, 5.53 mg OTA/kg b.w. caused no alterations of TBARS levels in the brain tissue of short-fin WT zebrafish. However, an increase in oxidative enzymatic activities, such as glutathione peroxidase, glutathione reductase, and glutathione-S-transferase, was observed by these authors, suggesting that this activation of oxidative defences prevented lipid peroxidation. Finally, Nogaim et al. (2020) reported that oral administration of 10 mg OTA/kg to rats resulted in a significant increase in MDA levels.

##### Measurement of the cellular GSH status

As mentioned before, GSH acts like an antioxidant, reacting directly with free radicals and also acting as a cofactor for some antioxidative enzymes. Therefore, the detection of GSH status can be used as an indicator of oxidative stress. Bhat et al. (2018) assessed the GSH levels by measuring its reaction with DTNB (Ellman’s reagent) and then carrying out the ABTS cation radical decolourisation assay (Re et al. 1999). These authors observed that i.p administration of 3.5 mg OTA/kg b.w. significantly reduced GSH content. The same result was obtained by Nogaim et al. (2020), who studied GSH status following Jollow et al. (1974) protocol, also based on its reaction with DNTB, after an oral administration of 10 mg OTA/kg b.w. to Balb/c mice.

##### Detection of superoxide (O2^−^) production

Superoxide dismutases (SOD) are enzymes that catalyse the dismutation of superoxide into O_2_ and H_2_O_2_. By measuring the activity of these enzymes, the production of O_2_^−^ can be specifically detected. Sava et al. (2006a), carried out a SOD assay following the procedure of Elstner & Heupel (1976), and observed that i.p administration of 3.5 mg OTA/kg b.w. caused a significant upregulation of SOD activity in brain tissue, which reached its peak at 24 h and returned to control levels by 72 h.

In contrast, authors such as Bhat et al. (2018) and Nogaim et al. (2020) detected that brain tissues from OTA-treated groups showed a decrease in SOD activity, compared to control groups. These authors (Bhat et al. 2018 and Nogaim et al. 2020) also assessed other oxidative stress markers. Bhat et al. (2018) measured glutathione reductase (GR) and glutathione peroxidase activities in brains from Balb/c mice i.p treated with 3.5 mg OTA/kg b.w. and found both to be decreased. Interestingly, Nogaim et al. (2020), also assayed GR (Carlberg & Mannervik 1985), but reported a significant increase in its activity using Wistar rats orally treated with 10 mg OTA/kg b.w.

In addition, they studied catalase (CAT) activity, by measuring the decline of an H_2_O_2_ solution by a spectrophotometer degradation method (Bhat et al. 2018), or by following Aebi’s protocol (Aebi 1984) (Nogaim et al. 2020). A significant increase in H_2_O_2_ degradation was detected in brain tissue by Bhat et al. (2018), after treatment with 3.5 mg OTA/kg b.w., indicating a decrease in CAT activity, while Nogaim et al. (2020) found CAT activity to be significantly increased in brain tissue of 10 mg OTA/kg b.w treated animals compared to control animals.

#### KE3: impaired proteostasis

Only one article has been found in the search studying the in vitro effects of OTA upon impaired proteostasis (Supplementary Table 5).

##### Monitoring of autophagy-related molecules

The chaperon-mediated autophagy (CMA) pathway is a highly specific degradation pathway for those cytosolic proteins (such as α-syn) with a KFREQ motif. Briefly, hsc70 recognises and binds to such motif and this complex is recognised by a lysosomal membrane receptor, LAMP-2A. Finally, the protein is internalised into the lysosome for its degradation. Izco et al. (2021), demonstrated, via WB techniques that oral treatment with 0.21 and 0.5 mg OTA/kg b.w. significantly decreased LAMP-2A protein levels, in a dose-dependent manner, while no changes in hsc70 levels were observed. Additionally, an increase in α-syn protein expression was detected (0.21 mg OTA/kg b.w.). All these alterations were observed in the midbrain tissue of Balb/c, 31 weeks after the end of the treatment (Table 3).

#### KE4: degeneration of dopaminergic neurons of the nigrostriatal pathway

DA cells in the SNpc and DA terminals in the striatum can be visualised using different phenotypic histological markers. Supplementary Table 6 presents all the information related to the degeneration of DA neurons from OTA in vivo studies, which is detailed further below, and summarised in Table 3.

##### Dopaminergic neurons in the striatum/SNpc

DA neurons can be detected by targeting the enzyme tyrosine hydroxylase (TH), that catalyses the conversion of L-tyrosine into L-DOPA (precursor of dopamine). Izco et al. (2021), determined the density of DA neurons via immunohistochemistry, identifying TH-positive neurons as DA neurons. After oral OTA treatment, a significant decrease in the number of TH-positive neurons was observed in the midbrain, as well as dose-dependent (0.21 and 0.5 mg OTA/kg b.w.) loss of striatal TH innervation.

Bhat et al. (2018), also studied TH, but focused on its activity in SNpc, striatum, hippocampus and whole brain, following the radiochemical assay described by Coyle & Axelrod (1972). TH activity was significantly reduced by i.p. OTA treatment in all brain structures (3.5 mg OTA/kg b.w.).

##### Dopamine content in the striatum/SNpc

Various modifications of the high-performance liquid chromatography (HPLC) technique were used by different authors in order to assess the dopamine content in the brain. Sava et al. (2006a) i.p. administered 0–6 mg OTA/kg b.w. to Swiss ICR mice and, employing HPLC with electrochemical detection to measure the levels of dopamine in the striatum, they reported a dose-dependent decrease in striatal dopamine. In a later study, Sava et al. (2006b) used the same technique to evaluate the effects of chronic OTA exposure (4, 8, 16 mg OTA/kg b.w.) over dopamine levels, via a subcutaneous continuous infusion (Alzet osotic minipumps). Dopamine content was found to be decreased after 2 cumulative doses of 8 mg OTA/kg b.w. Dopamine turnover was also determined, studying the levels of its metabolites 3,4 dihydroxyphenylacetic acid (DOVAC) and HVA (homovanillic acid) via HPLC, and calculating the ratio (DOVAC + HVA)/ Dopamine. This ratio was significantly increased after 2 weeks of OTA treatment.

Other authors (Bhat et al. 2018), estimated the levels of dopamine by reverse-phase HPLC technique, also coupled to an electrochemical detector. After 3 days of p.o administration of 3.5 mg OTA/kg b.w., dopamine levels in the striatum were found to be decreased, as well as in the hippocampus and whole brain tissue. Levels of DOPAV and HVA were also studied and a decrease was observed for both dopamine metabolites in these three brain structures.

##### Detection of Lewy Bodies (α-syn aggregates)

Through immunohistochemical techniques, Izco et al. (2021), detected the presence of phospho-α-syn aggregates in the midbrain of OTA-orally-treated mice (0.21 and 0.5 mg OTA/kg b.w.). Additionally, an increase (20% vs control) in the levels of S129 phosphorylated α-syn in the midbrain was detected by WB.

#### KE5: neuroinflammation

Only one article has been found in the search studying the in vivo effects of OTA upon neuroinflammation (Supplementary Table 7, Table 3).

##### Detection of astrocyte markers

Mateo et al. (2022) carried out immunofluorescent staining against GFAP to determine the effect of i.p. administered OTA over astrocytes in the hippocampus of male C57BL/6 mice. A dose-dependent decrease in GFAP-positive cells was observed, being 60% after 6 cumulative doses of 3.5 mg OTA/kg b.w. These authors also reported that treatment with OTA resulted in a morphological change of astrocytes, decreasing the number and length of cellular branches.

#### Adverse Outcome: parkinsonian motor deficits

Motor deficits are considered the adverse outcome of this AOP, and they can be evaluated by several behavioural tests. Supplementary Table 8 presents all the information related to the evaluation of parkinsonian motor deficits from OTA in vivo studies, which is detailed further below and summarised in Table 3.

##### Behavioural tests

Several tests can be carried out to study the behaviour of OTA-treated animals. El-Sayed et al. (2009) conducted a follow-up on the behaviour and swimming patterns of sea bass, which were orally treated with 0.05, 0.1, 0.15, 0.2, 0.25, 0.3, 0.35, and 0.4 mg OTA/kg b.w. Behavioural changes that could be identified as neurotoxic symptoms were reported. Other authors, such as Bhat et al. (2018) carried out a variety of specific behavioural tests to assess mice conduct. Gait analysis is used to study abnormalities in locomotion; the spontaneous activity test, more often called cylinder test, assesses the spontaneous forelimb as a way to estimate the sensory-motor function; the adhesive removal test, also known as tape test, evaluates a correct sensitivity and dexterity to remove an adhesive; the parallel bars test studies locomotor coordination; and the pole test is commonly used to assess basal ganglia-related disorders. Using all these tests, Bhat et al. (2018) observed a decrease in motor abilities in mice i.p. treated with 3.5 mg OTA/kg b.w. Similarly, Izco et al. (2021) detected a decrease in motor performance in mice orally treated with 0.21 and 0.5 mg OTA/kg b.w., using the wire hanging test, used to identify neuromuscular abnormalities of muscle strength and negative geotaxis test, which studies sensory and proprioceptive function. On the other hand, Valadas et al. (2023) adjusted the open field test to assess the motor behaviour of zebrafish, naming it as an open tank test and measuring parameters such as distance, crossings, absolute turn angle, mean speed, freezing episodes, and freezing duration. A decrease in distance, absolute turn angle and speed, and an increase in freezing time was detected in animals treated i.p. with 1.38, 2.77, 5.53 mg OTA /kg b.w. The social interaction test was also carried out, but no alterations in social behaviour were observed.

#### In vivo studies non-related to any KE of the AOP

For 6 in vivo, articles, published between 1987 and 2010, no relationship with any of the key events of the AOP could be established. As explained for in vitro articles, these studies were retained as they provide complementary insights into the neurotoxic mechanisms of OTA, which could inform about alternative or indirect pathways relevant to PD pathology. These 6 articles are presented in Supplementary Table 9. In 1 of these articles, Žanić-Grubišić et al. (1996) determined the activity of different enzymes via different assays. They concluded that 0.120 mg OTA/kg b.w., administered via gastric intubation to Fischer rats, caused an increase in the activity of membrane-bound enzymes (Ecto-Ca^2+^/Mg^2+^ ATPase, alanine aminopeptidase, ecto-5'Nucleotidase and γ-glutamyl transferase) and even a larger increase in cytosolic and lysosomal enzymes (lactate dehydrogenase and N-acetyl-β-D-glucosaminidase). However, they observed that this increase in activity returned to control levels over the course of the days, despite continuing with the treatment.

In another 2 of these 6 articles, Belmadani et al. (1998a) and Belmadani et al. (1998b) measured the levels of free amino acids in the brain via HPLC. While Belmadani et al. (1998a) administered 0.289 mg OTA/kg b.w. via gastric intubation to Wistar rats, Belmadani et al. (1998b) treated Wistar rats with stereotaxic injections of 403 ng OTA/10 μL of NaCl solution. Both detected a decrease in general levels (i.e. tyrosine) of amino acids but an increase in the levels of phenylalanine. Belmadani et al. (1998b) also determined the enzymatic activities of lactate dehydrogenase (LDH) and deoxyribonuclease (DNase), both of which were found to be increased in central mesencephalon after OTA treatment. The activity of DNase was also increased in the rest of the brain tissue. Besides, they performed a separate study, treating Wistar rats with oral doses of 0.289 mg OTA/kg b.w. for 8 days, and determined the enzymatic activities of LDH and DNase. The activity of LDH was found to be increased in the striatum, hippocampus, mesencephalon, and cerebellum, while DNase’s activity was increased in mesencephalon and cerebellum. On the other hand, Belmadani et al. (1998a) carried out a histopathology analysis that showed a growth in the number of pyknotic cells in the hippocampus. Other authors, (Mantle & Nolan 2010), also performed a histopathology analysis, finding the presence of large eosinophilic bodies in the brainstem of Fischer rats orally treated with 5 ppm OTA, although this was thought to be related to aging rather than to OTA treatment. In another histological analysis performed by Dortant et al. (2001), an increase in lesions (cerebellar white-matter vacuolation) was observed in the brains of OTA-treated SPF Wag/mbl rats, and was considered not to be artefact-related, as it was a dose-dependent increase (oral administration of 0.07, 0.34, 1.68 mg OTA/kg b.w.). Finally, Delibas et al. (2003) used WB techniques to study whether OTA oral treatment has any effect upon hippocampal glutamate receptor (NMDAR) subunits 2A and 2B. They reported that oral administration of 0.289 mg OTA/kg b.w. to Wistar rats reduced the expression of these proteins (Supplementary Table 9).

## Discussion

With the aim of evaluating OTA neurotoxicity mechanisms and their potential link to neurodegenerative diseases such as PD, a systematic review of scientific articles studying OTA’s neurotoxic effects was carried out. Of the 545 articles initially retrieved, 515 were excluded, often for more than one reason. The main exclusion criteria were studies not focused on neurotoxicity (primarily addressing nephrotoxicity) or those centred on neurodevelopmental effects. A considerable number of review articles were also excluded, often overlapping with other exclusion criteria, significantly contributing to the high exclusion rate. No filter for the timeline was applied, which contributed to the large number of retrieved articles. Moreover, the search terms were designed to be inclusive, combining ‘OTA AND (… OR …),’ without applying exclusionary terms like ‘NOT,’ to ensure the retrieval of all articles that addressed neurotoxicity, directly or indirectly. Moreover, the selected keywords for the search (such as ‘mitochondria,’ ‘oxidative stress,’ and ‘bioenergetic’) were known to be broad and not specific for neurotoxicity. However, they were intentionally selected because they are linked to key events described in the AOP for PD, ensuring to include all studies assessing OTA's potential contribution to PD.

To determine whether these studies address endpoints relevant to PD, the retrieved articles were analysed according to the OECD AOP for PD. None of the retrieved articles studied the MIE (Binding of an inhibitor to Complex I). The mechanism of action of this mycotoxin has not been fully described and seems to be very complex (Kőszegi & Poór, 2016). Although the inhibition of Complex I by other neurotoxins, such as rotenone, has been demonstrated (Degli Esposti 1998), little evidence of OTA binding to NADH dehydrogenase can be found. On a similar note, none of the reviewed articles studied KE1 (inhibition of Complex I). Other researchers studied the effects of OTA over different cell lines: Schwerdt et al. (2021) observed that exposure of renal cells and fibroblasts to OTA decreased the expression of NDUFB10, a gene that encodes for mitochondrial NADH ubiquinone oxidoreductase subunit B10, (part of the mitochondrial respiratory Complex I). However, there is no evidence in this matter using neuronal models. Consequently, there is no evidence addressing the impact of OTA on the initial KEs of the PD AOP in neuronal models.

On the other hand, KE2 (mitochondrial dysfunction) was the most assessed endpoint among in vitro and in vivo studies. Mitochondrial dysfunction is a consequence of the inhibition of the mitochondrial respiratory chain and the subsequent ROS generation and oxidative stress (Bal-Price et al. 2018). The reviewed in vitro studies employed OTA concentrations ranging from 0.1–150 µM, consistently reporting dose-dependent increases in mitochondrial dysfunction biomarkers. These findings align with short- and mid-term in vivo studies, which employed OTA doses ranging from 3.5–6 mg OTA/kg b.w. i.p. in mice and 10 mg OTA/kg b.w. orally in rats, consistently showing mitochondrial dysfunction in both species. For example, all authors that assessed ROS generation (Babayan et al. 2020; Baldi et al. 2004; Bhat et al. 2016; Yoon et al. 2009) agreed that OTA causes an increase in ROS production in different neuronal models. OTA-induced oxidative stress was also indirectly detected in animal models, measuring the activity of the enzyme OGG1 (Sava et al. 2006a). These authors also stated that oxidative DNA damage was determined by a standard comet assay. However, the standard version of this technique only allows to detect DNA breaks and AP (apurinic/apyrimidinic) sites. To detect oxidative DNA damage, a modification of the comet assay must be applied, such as the Fpg-comet assay. All retrieved articles evaluating KE2 in vitro (Bhat et al. 2016; Park et al. 2019; Zhang et al. 2009) agree on the fact that OTA alters other parameters of mitochondrial dysfunction: decrease of ∆Ψm and the MPTPo (indicated by an increase of mitochondrial Ca^++^ ions). In vitro (Belmadani et al. 1999; Bhat et al. 2016) and in vivo (Bhat et al. 2018; Nogaim et al. 2020; Sava et al. 2006a) studies showed that OTA induced lipid peroxidation (increase in MDA levels). Only 1 article (Valadas et al. 2023) reported no effect of OTA treatment over MDA levels, being this the only in vivo study using a non-rodent experimental model (zebrafish). This fact highlights potential species-specific differences in OTA sensitivity regarding neurotoxicity. Considering the assessment of superoxide production, Bhat et al. (2018) and Nogaim et al. (2020) reported a decrease in SOD activity in brain tissue, while Sava et al. (2006a) detected an upregulation followed by a return to control levels. This could be explained by focusing on the duration of the treatment. Mice were treated with OTA for 3 days by Bhat et al. (2018) and for 28 days by Nogaim et al. (2020), while Sava et al. (2006a) administered a single OTA dose and observed the effects at 6 h, 24 h, and 72 h after the administration. At 24 h, they observed a peak in the upregulation of SOD activity, decreasing down to control levels at 72 h. Sava et al. (2006a) results could align with Bhat et al. (2018) and Nogaim et al. (2020) observations: OTA could be inducing ROS production since the beginning of the treatment, triggering a defence response in the cell (short-term upregulation of SOD activity), which would fail with time, resulting in a long-term decrease of SOD activity. Bhat et al. (2018) and Nogaim et al. (2020) also reported a reduction in GSH levels, which is a tripeptide that can serve as a cofactor for some antioxidant enzymes, supporting the hypothesis that OTA causes an impairment on the antioxidative system.

Thus, several points can be highlighted. Firstly, various techniques are being currently employed to assess different endpoints of KE2 of the AOP of PD, being this KE widely covered. Secondly, a consensus can be reached through all retrieved articles evaluating this KE, stating that OTA causes mitochondrial dysfunction at neuronal (in vitro) and brain (in vivo) level, by enhancing ROS production, decreasing ∆Ψm, promoting MPTPo and lipid peroxidation and impairing the antioxidative enzymatic system.

Only 1 article studied KE3 (impaired proteostasis). Izco et al. (2021) assessed this KE in vitro using concentrations of 0.1 and 0.2 µM OTA, revealing a significant increase in α-syn half-life and a reduction in LAMP-2A levels after 72 h of treatment. These findings were corroborated in vivo, where oral doses of 0.21 and 0.5 mg OTA/kg b.w. similarly reduced LAMP-2A protein levels and increased α-syn aggregation, 6 months after the end of the treatment. This highlights how little is currently known about OTA’s effects on autophagy from a neurotoxic perspective. Although other studies have explored OTA’s influence on autophagy, these have mostly focused on nephrotoxicity (Khoi et al. 2021). Addressing this gap in the context of neurotoxicity is essential to better understand OTA’s potential involvement in PD-related mechanisms.

Regarding KE4 (degeneration of DA neurons of the nigrostriatal pathway), only in vivo articles were found to address its endpoints. Doses ranging from 3.5 to 6 mg OTA/kg b.w. i.p., 4–16 mg OTA/kg b.w. subcutaneously, and 0.21 and 0.5 mg OTA/kg b.w. orally in mice consistently led to DA alterations. When evaluating dopamine content in different brain structures, all authors (Bhat et al. 2018; Sava et al. 2006a, 2006b) agreed on a decrease in dopamine levels after OTA administration. On the other hand, Izco et al. (2021) reported a decrease in TH + DA neurons in midbrain, loss of DA innervation in the striatum, and the presence of p-syn aggregates in the midbrain. All together indicates that OTA exposure results in the neurodegeneration of DA cells in the nigrostriatal pathway.

Since no articles were retrieved studying this KE through in vitro techniques, this could be thought of as a gap in current in vitro methods and strategies for the evaluation of neurotoxicity. Developing new approach methodologies (NAMs) that can address these endpoints in a mechanistically relevant manner is crucial for reducing reliance on in vivo studies and advancing the field of neurotoxicity testing. In vitro approaches, such as the use of advanced human cell models or organoids combined with imaging techniques for the detection of p-syn aggregates (Volpicelli-Daley et al. 2014) could provide valuable insights. However, such models may currently lack the complexity needed to fully replicate the interaction of DA neurons with the surrounding cellular and molecular environment. Addressing these limitations through the integration of emerging technologies, including NAMs, could help fill this gap and improve the understanding of OTA’s neurodegenerative potential, particularly its role in the degeneration of DA neurons.

The study of KE5 (neuroinflammation) has expanded considerably in the last decade. It has been reported that the degree of activation of microglial cells has a correlation with the degree of DA terminal loss in PD (Ouchi et al. 2005). One of the main glial cells in the central nervous system is astrocytes (Jessen 2004), having been reactive astrocytes detected in the SNpc of PD patients (Miklossy et al. 2006). Quantification of GFAP levels is one of the most common techniques to evaluate the activation of astrocytes in response to brain damage (astrogliosis). In general, GFAP levels are increased following astrocytic activation. However, contradictory outcomes have been reported regarding GFAP levels in PD. Damier et al. (1993) described no differences in GFAP staining in different brain areas of control and PD patients, while Thannickal et al. (2007) observed increasing levels of GFAP with PD’s progression in the hypothalamus of PD cases compared to controls. Regarding OTA effects over GFAP expression, authors of all retrieved articles assessing neuroinflammation (Mateo et al. 2022; Razafimanjato et al. 2010; Von Tobel et al. 2014; Zurich et al. 2005) reported a decrease in GFAP levels after exposure to the mycotoxin, using OTA concentrations ranges of 0.01–10 µM for in vitro studies and a i.p. dose of 3.5 mg OTA/kg b.w. in mice. Interestingly, Zurich et al. (2005) detected a dose-dependent (0.01 and 0.02 µM OTA) increase in vimentin levels, which is another intermediate filament protein, that provides, together with GFAP, structural support to astrocytes, and its expression is usually enhanced during astrogliosis (O’Leary et al. 2020). Some studies suggest that in the initial stages of neurodegenerative processes, astrocytes are activated, which results in an increase of astrocytic markers such as GFAP and vimentin (Voronkov et al. 2023). In contrast, later stages of neurodegeneration show a decrease in GFAP expression (Voronkov et al. 2023), which could be due to astrocyte senescence. Indeed, Mateo et al. (2022) reported that treatment with 3.5 mg OTA/kg b.w. resulted in a morphological change of astrocytes, decreasing the number and length of cellular branches. Interestingly, Razafimanjato et al. (2010) tested OTA concentrations 1000 times higher than those used by Zurich et al. (2005) (0.01 µM vs 10 µM), when studying alterations in astrocytical activity, reporting similar results. Both authors found GS activity and expression to be downregulated by OTA, indicating a decrease in the activity (glutamate intake) of astrocytes. These findings suggest that OTA's effects on astrocytic activity are robust across a broad range of doses, highlighting its potent neurotoxic effects on astrocytes. On the other hand, Von Tobel et al. (2014) also observed a OTA-dependent depletion in metallothioneins I and II (MTI and MTII) levels, using low concentrations of OTA (0.01 µM). MTI and MTII are molecules with antioxidant and metal-binding properties (Ebadi et al. 2005). In contrast to Von Tobel et al. (2014) results, Michael et al. (2011) reported MTI and MTII levels to be upregulated in astrocytes in SNpc of PD patients, and Miyazaki et al. (2011) demonstrated that excessive production of dopamine induces the expression of MTI and MTII in striatal astrocytes and MTs, in turn, exert DA neuroprotective properties when exposing cells to a neurotoxin (quinone), due to its quinone-quenching ability. All this indicates that MTI and MTII may be somehow involved in OTA-induced neuroinflammation and that the mycotoxin alters their levels, affecting the ability of the glia system to protect neurons against OTA neurotoxic effects.

Neuroinflammation can be neuroprotective or neurodegenerative (Monnet-Tschudi et al. 2007), being characterised by M2 and M1 neuroinflammatory phenotype, respectively (Kigerl et al. 2009). Von Tobel et al. (2014) observed a general increase in microglial activation after OTA exposure, indicated by an increase in IB4 labelling. Then, they demonstrated that long-term exposures (10 days) to 0.01 µM OTA induce the expression of M1 neurodegenerative phenotype in microglia. That is, upregulation of pro-inflammatory cytokines IL-6, IL-1ß, and TNF-α levels, and downregulation of anti-inflammatory cytokine IL-4 levels. Interestingly, these authors showed that shorter exposures to 0.01 µM OTA (48 h) increased M1 phenotypical markers mRNA levels (Itgam and cd86), but these markers were not detected by immunolabelling until 10-day repeated exposure. This indicated that OTA favours an early M1 activation, but it needs a longer time to be fully activated. However, the fact that not all IB4-labelled cells expressed M2 phenotypes, and the increased Mcr1 mRNA levels, indicated that OTA predominantly triggered an M1 response, but also induced a less pronounced M2 response, coexisting both phenotypes. The activation of neurodegenerative M2 inflammatory phenotype by OTA was also reported by other researchers (Chansawhang et al. 2022; Penalva-Olcina et al. 2024). These authors observed M1 activation after shorter OTA exposures, but higher OTA doses (0.05–12.5 µM). Moreover, Chansawhang et al. (2022) suggested that 0.05–2 µM OTA activates microglia via ERK and p38 MAPK pathways in a dose-dependent way. On the other hand, Penalva-Olcina et al. (2024) also reported an increased IL-6 and TNF-α production after OTA treatment (3.1, 6.25, 12.5 µM), but they observed these alterations in neuroblastoma SH-SY5Y cells, and not in glial model cells. They concluded that this increase in pro-inflammatory cytokines may be related to the OTA-induced neurotoxicity and the cell cycle alterations they observed.

Therefore, we can conclude that OTA triggers neuroinflammation, affecting astrocytes and microglial cells. It could be hypothesised that OTA-induced neuroinflammation is mainly neurodegenerative (M2 phenotype) and that in later stages it could be causing astrocytic senescence, and that glial neuroprotective ability could be compromised by OTA exposure, leading to neurotoxicity. The consistent observation of neuroinflammatory effects across varied experimental conditions, despite differences in concentration ranges, highlights the capacity of OTA to disrupt neuroinflammatory pathways. The studies reviewed encompass a wide range of OTA concentrations, from low, potentially relevant to real-world exposures, to higher doses often employed to observe acute toxic effects. With all this in mind, it can be said that K5, neuroinflammation, is being widely studied in vitro, while only 1 in vivo study assessed OTA neuroinflammatory effects. Further research with in vivo models might be needed to deepen in OTA neuroinflammatory profile.

Finally, the adverse outcome of this AOP was evaluated by some authors. All described techniques for the assessment of this endpoint can only be applied to in vivo models. Indeed, all retrieved articles studying the adverse outcome were in vivo studies. Different behavioural tests were applied to evaluate parkinsonian motor deficits by different authors. Bhat et al. (2018) and Izco et al. (2021) employed the mouse model Balb/c, and applied behavioural tests for rodent models such as gait analysis, spontaneous activity, adhesive removal, parallel bars (Bhat et al. 2018), wire hang test and negative geotaxis test (Izco et al. 2021). Both authors observed a deterioration in mice's motor performance after OTA administration. On the other hand, El-Sayed et al. (2009) and Valadas et al. (2023) used fish models (sea bass and zebrafish, respectively). Both conducted a follow-up of swimming patterns, reporting locomotor impairment in both models. Valadas et al. (2023) also performed a social interaction test, but no alterations in social behaviour were observed. El-Sayed et al. (2009) observed muscular seizures occurring in sea bass before death. The OTA doses used in these studies varied widely, from low concentrations and prolonged exposure (e.g. Izco et al. (2021): 0.21–0.5 mg OTA/kg b.w. p.o.), to higher doses designed for acute effects (e.g. Bhat et al. (2018): 3.5 mg OTA/kg b.w. p.o.).

All this indicates that OTA induces behavioural alterations, related to an impairment of locomotor abilities in different animal models. OTA-induced parkinsonian AO can be also assessed by non-invasive imaging of DA neuron terminals, although this endpoint is not evaluated in any retrieved article. Imaging of DA terminals could be performed both in vivo and ex vivo, giving valuable insight on any alterations on the morphology and function of DA neurons after OTA treatment. Evaluation of this endpoint ex vivo has the advantage of reduced animal use, compared to in vivo studies, although it has its limitations, such as the loss of physiological context, or the studying of complex behaviours, limitations that can be overcome with in vivo models.

The ability of OTA to affect the Central Nervous System  (CNS) is not fully understood, partly because of uncertainties regarding its ability to cross the blood–brain barrier (BBB). Computational predictions, such as those from SwissADME tool (Daina et al. 2017) suggest that OTA has high intestinal absorption but limited capacity to permeate the BBB. In line with this, Behrens et al. (2021) demonstrated through in vitro studies that 10 μM OTA showed cytotoxic effects on porcine brain capillary endothelial cells (PVCBE) and 1 μM OTA exerted barrier-weakening effects on these cells. These results were evidenced by a reduction in transendothelial electrical resistance (TEER) and an increase in sucrose permeability. They also demonstrated that OTA was able to cross an in vitro BBB model, but the amount of the mycotoxin transferred to the brain compartment was low, comparable to the amounts of 14C-sucrose, a negative permeability marker, which is known not to cross the BBB in vivo. Based on these findings, they concluded that OTA was unlikely to permeate the BBB under normal in vivo conditions. In relation to in vivo studies in which OTA was detected in the brain, the authors of the above-mentioned in vitro study hypothesised that the barrier-weakening effect of OTA might have allowed the influx of potentially harmful compounds including OTA itself to the brain and explained the in vivo findings. Indeed in vivo studies have provided evidence that OTA is indeed able to reach the CNS. Using HPLC techniques, authors like Belmadani et al., (1998a), Belmadani et al., (1998b) and Sava et al., (2006a) were able to detect the mycotoxin in specific brain regions. More recently, Izco et al. (2021) orally administered 0.21, 0.5, 1.5, and 4.5 mg OTA/kg b.w. to Balb/c mice for 28 days, detecting levels of OTA in plasma with all tested doses. Following the oral administration of 1.5 mg OTA/kg b.w. OTA concentrations in plasma ranged from 3708 to 5944 µg/L, whereas brain concentrations ranged from 20 to 110 ng/g. Notably, OTA levels in the brain were below the limit of quantification (LOQ) after treatment with 0.21 and 0.5 mg OTA/kg b.w. A similar in vivo study was carried out by Beraza et al. 2024, although doses of 0.21 or 0.5 mg OTA/kg b.w. were administered i.p. instead of p.o. They reported a mean OTA concentration of 3.19 ± 0.87 ng/g in the brain after treatment with the low dose (0.21 OTA/kg b.w.), being approximately 276 times higher in plasma (880.45 ± 121.61 ng/ml) and 16 times higher in kidney (51.14 ± 12.70 ng/g). Regarding the 0.5 mg OTA/kg b.w. dose, by Beraza et al. (2024) reported a mean OTA concentration of  9.27 ± 2.89 ng/g in the brain, being approximately 294 times higher in plasma (2853.06 ± 812.59 ng/ml) and 15 times higher in the kidney (138.16 ± 30.77 ng/g). These studies demonstrate that, while its permeability might be limited, OTA is able to reach the CNS, potentially accumulating over time or under repeated exposure. Also, the comparison between these studies highlights the importance of considering administration routes when evaluating OTA's toxicokinetics and its potential to impact the CNS. Finally, it is important to note that OTA potential role in the development of PD might not have to implicate a direct effect on the CNS. It has been hypothesised that PD pathology could be initiated in the enteric nervous system and be propagated in a prion-like manner from there to the CNS through the dorsal motor nucleus of the vagal nerves (Braak & Del Tredici 2008). Taking this hypothesis into account, the role of OTA in PD pathology would not necessarily require the toxin to cross the BBB, as its effects on the enteric nervous system could initiate a cascade of events leading to neurodegeneration in the CNS.

The concentrations of OTA tested in all aforementioned studies should be interpreted in the context of real-world exposure levels. Human plasma concentrations of OTA have been reported to range from 0.2 to 10 ng/mL (EFSA 2020), which corresponds to approximately 0.0005–0.025 µM. While many in vitro studies employed concentrations considerably exceeding this range (e.g., 0.1–150 µM OTA in studies assessing KE2), other studies used concentrations closer to those observed in humans. For instance, Zurich et al. (2005) used 0.01–0.02 µM OTA to study astrocytic activity and reported significant alterations in GFAP and vimentin expression, findings that aligned with those observed at much higher concentrations (e.g., 10 µM OTA in Razafimanjato et al. 2010). Regarding human dietary exposure, EFSA has established the average intake of OTA to be approximately 0.015–0.04 µg/kg b.w./day for the general population, with higher estimates of up to 0.1 µg/kg b.w./day for high consumers. While the doses used in many in vivo studies, such as 3.5–6 mg/kg b.w. i.p. in rodents (Bhat et al. 2018; Nogaim et al. 2020), are far higher than dietary intake, studies like Izco et al. (2021) employed lower oral doses of 0.21–0.5 mg/kg b.w. OTA over 28 days, which better mimic potential human exposure scenarios. However, even these doses exceed typical dietary intake levels, reflecting the challenges in designing animal studies that balance the need for observable outcomes with relevance to real-world exposures. To provide regulatory context, EFSA has also established a Margin of Exposure (MoE) for OTA, based on a Benchmark Dose Lower Confidence Limit (BMDL10) of 4.73 µg/kg b.w./day for non-neoplastic effects, specifically nephrotoxicity in pigs. Current MoE calculations raise concerns for high consumers and vulnerable populations, such as children, who might be exposed to dietary OTA at levels approaching this threshold. The doses employed in many of the reviewed in vivo studies, such as those by Bhat et al. (2018) and Nogaim et al. (2020) were significantly higher than the BMDL10, with doses ranging from 3.5 to 6 mg OTA/kg b.w. However, the BMDL10 assumes lifetime daily exposure, making direct comparisons challenging.

In conclusion, this review highlights significant research gaps in understanding OTA’s neurotoxicity, particularly its role in neurodegenerative pathways linked to PD. While mitochondrial dysfunction (KE2) is well-studied and consistently linked to OTA exposure, early key events (MIE: Complex I binding and KE1: Complex I inhibition) and mechanisms related to impaired proteostasis (KE3) remain largely unexplored in neuronal models. Evidence supports OTA's contribution to later key events, such as neuroinflammation (KE5) and dopaminergic neuron degeneration (KE4), promoting neurodegenerative pathways. Addressing these gaps, particularly in early mitochondrial interactions and autophagy-related mechanisms, is crucial for advancing the understanding of OTA-induced neurotoxicity and its relevance to PD.

Although this review focuses on the neurotoxic effects of OTA and its potential link to neurodegenerative diseases, specifically PD, it is worth noting that other mycotoxins have also been reported to interfere with the CNS. For instance, aflatoxins and fumonisins have been associated with oxidative stress and neuronal damage, which may contribute to the development of neurological disorders, as highlighted in studies such as Lee et al. (2017). These scattered findings underscore the broader potential role of foodborne and environmental mycotoxins in the etiology of degenerative neurological diseases, an area that needs further research. While our review specifically addresses OTA, acknowledging the diversity of mycotoxin exposure and its possible CNS effects provides an additional context for understanding the underestimated impact of these contaminants on public health.

## Supplementary Information

Below is the link to the electronic supplementary material.

Supplementary file1 (PDF 205 KB)
