# Supplementary material for: Potential role of ochratoxin A in Parkinson’s disease: a systematic review of current evidence
Source: Arch Toxicol. 2025 Mar 6;99(5):1769–90. doi: 10.1007/s00204-025-03994-5 (PMC12085323; doi:10.1007/s00204-025-03994-5)
Supplement: Supplementary file 1 — Supplementary file1 (PDF 205 KB) [file 204_2025_3994_MOESM1_ESM.pdf]

Supplementary Table 1 Data extraction of reviewed articles assessing **KE2: mitochondrial dysfunction by *in vitro* techniques**.

**KE2: mitochondrial dysfunction**				
**Cell line**	**[OTA]/Exposure time**	**Assay**	**Result**	**Reference**
Intracellular ROS direct analysis				
Human neuroblastoma (SK-N-MC)	12.38, 24.76 and 49.53 µM / 24, 48 h	DCFH-DA staining assay	Dose-dependent increase of ROS at both times	Baldi et al., 2004
Human neuroblastoma (SH-SH5Y)<br>Mouse neural cells (HT22)	100 µM / 30 min – 1 h	DCFH-DA staining assay	Increase of ROS in both cell lines at both times	Yoon et al., 2009
Mouse neural cells (Neuro-2a)	100, 250 and 500 nM / 24 h	DCFH-DA staining assay	Significant dose-dependent increase of ROS	Bhat et al., 2016
Human neuroblastoma (SH-SH5Y)<br>Mouse neural cells (HT22)	10 µM / 30 min – 6 h	Dihydroethidium (DHE) staining assay	SH-SY5Y: significant increase of ROS after 30 min, which remained unchanged during 6h<br>HT22: significant increase of ROS after 30 min, which gradually decreased during 6h	Babayan et al., 2020
Retinal ganglion cells (RGC-5)	248 and 496 nM / 3 days	ROS detection kit (Jiancheng, Nanjing, China)	Significant dose-dependent increase of ROS	Fu *et al*., 2024
Gibco® Human Astrocyte (GHA)	5-15 µM / 24 h	DCFH-DA staining assay	Significant dose-dependent increase of ROS	Chu *et al*., 2024
Intracellular ROS indirect analysis				
Human neuroblastoma (SH-SH5Y)<br>Mouse neural cells (HT22)	10 µM / 30 min – 72 h	Fpg-comet assay	SH-SY5Y: maximal level of Fpg-sensitive sites detected after 1 h OTA treatment, and significantly decreased after 72 h<br>HT22: maximal level of Fpg-sensitive sites detected after 1 h OTA treatment, and significantly decreased after 24 h	Babayan et al., 2020
Quantification of lipid peroxidation				
Primary rat neurons and astrocytes	10, 20, 25, 50, 75, 100, 150 µM / 46 h	MDA detection	Dose-dependent MDA increase in both cell lines	Belmadani *et al*., 1999
Mouse neural cells (Neuro-2a)	100, 250 and 500 nM / 24 h	MDA detection	Significant MDA increase at 250 and 500 nM OTA	Bhat *et al*., 2016
Retinal ganglion cells (RGC-5)	248 and 496 nM / 3 days	MDA detection	Significant dose-dependent increase of MDA	Fu *et al*., 2024
Measurement of the cellular GSH status				
Retinal ganglion cells (RGC-5)	248 and 496 nM / 3 days	GST content assay (Jiancheng, Nanjing, China)	Dose-dependent decrease of GST levels	Fu *et al*., 2024
Gibco® Human Astrocyte (GHA)	5-15 µM / 24 h	CMFDA staining assay	GSH levels were significantly reduced in a dose-dependent manner	Chu *et al*., 2024
Detection of superoxide production				
Retinal ganglion cells (RGC-5)	248 and 496 nM / 3 days	SOD detection kit (Jiancheng, Nanjing, China)	SOD levels were significantly decreased at both concentrations	Fu *et al*., 2024
Measurement of Mitochondrial Membrane Potential (ΔΨm)				
Retinal ganglion cells (RGC-5)	248 and 496 nM / 3 days	JC-1 staining	Significant loss of Δψm in OTA-exposed cells	Fu *et al*., 2024
Human neuroblastoma (SH-SH5Y)<br>Primary rat neurons	0.1, 0.25, 1.0 and 2.5 µM / 8 h	JC-1 staining	Dose-dependent loss of Δψm in both cell lines	Zhang *et al*., 2009
Mouse neural cells (Neuro-2a)	100, 250 and 500 nM / 24 h	Rhodamine 123 staining	Dose-dependent loss of Δψm	Bhat *et al*., 2016
Normal Human Astrocytes (NHA-SV40LT)	0.5, 1 and 2 µM / 48 h	JC-1 staining	Significant loss of Δψm at 2 µM OTA	Park *et al*., 2019
Mitochondrial permeability transition pore opening (MPTPo) evaluation				
Normal Human Astrocytes (NHA-SV40LT)	0.5, 1 and 2 µM / 48 h	Fluo-4 staining (cytosolic Ca^{++}) and Rho-2 staining (mitochondrial Ca^{++})	Slight increase in the number of intracellular Ca^{++} ions<br>Significant increase of mitochondrial Ca^{++} at 2 µM OTA	Park *et al*., 2019

CMFDA: 5-chloromethyl fluorescein diacetate; DCFH-DA: dichlorofluorescin diacetate; DHE: dihydroethidium; GST: glutathione-S-transferase; GSH: gluthathione; MDA: malondialdehyde; MPTPo: mitochondrial permeability transition pore opening; OTA: Ochratoxin A; ROS: Reactive Oxygen Species; SOD: superoxide; [OTA]: Ochratoxin A concentration; ΔΨm: mitochondrial membrane potential.

Supplementary Table 2 Data extraction of reviewed articles assessing **KE3: impaired proteostasis by *in vitro* techniques**.

**KE3: impaired proteostasis**				
**Cell line**	**[OTA]/Exposure time**	**Assay**	**Result**	**Reference**
General turnover assays				
WT α-syn SH-SH5Y	100 nM / 72 h	Standard cycloheximide procedure (WB)	OTA treatment significantly increased α-syn half-life (by 26%)	Izco *et al*., 2021
	100 and 200 nM / 72 h	WB and RT-qPCR	100 and 200 nM OTA significantly decrease LAMP-2A protein levels, while mRNA expression was downregulated only by 200 nM OTA. No changes in hsc70 expression were observed.	
Monitory of autophagy-related molecules				
WT α-syn SH-SH5Y	100 and 200 nM / 72 h	WB and RT-qPCR	100 and 200 nM OTA significantly decrease LAMP-2A protein levels, while mRNA expression was downregulated only by 200 nM OTA. No changes in hsc70 expression were observed.	Izco *et al*., 2021

Hsc70: heat shock cognate protein 70; LAMP-2A: lysosome-associated membrane protein 2A; OTA: Ochratoxin A; RT-qPCR: quantitative Reverse Transcription Polymerase Chain Reaction; WB: Western Blot; α-syn: alpha synuclein; [OTA]: Ochratoxin A concentration.

Supplementary Table 3 Data extraction of reviewed articles assessing **KE5: neuroinflammation by *in vitro* techniques**.

**KE5: neuroinflammation**				
**Cell line**	**[OTA]/Exposure time**	**Assay**	**Result**	**Reference**
Detection of astrocyte markers				
Primary rat astrocytes	10 and 20 nM / 24, 48 h	RT-qPCR and biotinylation techniques	GFAP: OTA reduced GFAP mRNA expression at 24 h and 48 h. GFAP staining was significantly diminished by OTA at 48 h.<br>Vimentin: dose-dependent increase of vimentin mRNA levels at 24 h and 48 h, while its intensity staining presented no change.	Zurich *et al*., 2005
Primary rat astrocytes	10 µM / 72 h	ELISA assay	No significant effect over GFAP and GLT-1 total expression	Razafimanjato *et al*., 2010
		Biotinylation techniques	50% decrease in the cell surface expression of GFAP and GLT-1	
Detection of astrocyte markers				
Primary rat astrocytes	10 nM / 48 h	Detection of GFAP and MTI/MTII: RT-qPCR and biotinylation techniques	Significant decrease of MT1/MTII expression after 48 h of exposure and a decrease of GFAP mRNA levels after 24 h of exposure.	von Tobel *et al*., 2014
Detection of microglial activation				
Oligodendrocytes and microglial cells	10 nM / 48 h and 10 days	Immunolabelling technique	Significant increase of IB4 positive cells after 10 days	von Tobel *et al*., 2014
		Quantification of cytokines expression (IL-4, IL-6, IL-1ß, TNF-α), M1 and M2 microglial phenotype (Itgam/Cd11b and Cd86/B7-2; Arg1 and Mrc1/Cd206) through RT-qPCR	Increased expression of pro-inflammatory cytokines and decreased levels of anti-inflammatory cytokines after 10 days<br>Rise of Itgam and Cd86 levels and upregulated Mrc1 expression at both timepoints.	
Glutamine synthetase (GS) assay				
Primary rat astrocytes	10 and 20 nM / 24, 48 h	Quantification of GS mRNA levels through RT-qPCR. Measurement of GS activity through a colourimetric method	20 nM OTA significantly decreased GS mRNA levels and GS activity 48 h post-treatment	Zurich *et al*., 2005
Glutamine synthetase (GS) assay				
Primary rat astrocytes	1, 10 and 100 µM / 72 h	Measurement of GS activity through a colourimetric method	100 µM OTA significantly inhibited GS activity	Razafimanjato *et al*., 2010
BV-2 cells	50-2,000 nM / 24 h	Quantification of cytokines expression (iNOS, IL-1ß, IL-6, TNF-α) through RT-qPCR; measurement of IL-6 by ELISA; determination of NO production by Griess reagent	A dose-dependent upregulation of IL-6, TNF-α, IL-1ß, and iNOS mRNA levels was observed after 24 h OTA exposure; as well as extracellular IL-6 and NO levels.	Chansawhang *et al*., 2022
Human neuroblastoma (SH-SH5Y)	3.1, 6.25, 12.5 µM<br>24 h and 48 h	ELISA assay	IL-6 and TNF-α expressions were slightly increased after 24 h OTA exposure and significantly increased after 48 h in all three doses.	Penalva-Olcina *et al*., 2024

Arg1: arginase 1; Cd86/B7-2: cluster of differentiation 86; GFAP: glial fibrillary acidic protein; GLT-1: glial glutamate transporter type 1; GS: glutamine synthetase; IL: interleukin; IB4: isolectin B4; iNOS: inducible nitric oxide synthase; Itgam/Cd11b: integrin alpha M; Mrc1/Cd206: mannose receptor C type 1; MTI/II: melatonin receptor type 1 and 2; NO: nitric oxide; OTA: ochratoxin A; RT-qPCR: quantitative reverse transcription polymerase chain reaction; TNF: tumour necrosis factor; [OTA]: ochratoxin A concentration.

Supplementary Table 4 Data extraction of reviewed articles assessing **KE2: mitochondrial dysfunction by *in vivo* techniques**.

**KE2: mitochondrial dysfunction**					
**Experimental system**	**OTA dose**	**Route/Administration period**	**Assay**	**Result**	**Reference**
Intracellular ROS indirect analysis					
Male Swiss ICR mice	0–6 mg/kg b.w.	i.p. single-dose<br>Observation 6, 24 and 72 h after administration	Standard comet assay	Oxidative DNA damage was increased across all brain regions at all time points, having its peak at 24 h, after administration 3.5 mg OTA/kg b.w.	Sava et al., 2006a
			Detection of OGG1 activity	Inhibition of OGG1 activity in all brain regions, having a peak at 6 h.	
Quantification of lipid peroxidation					
Male Swiss ICR mice	0–6 mg/kg b.w.	i.p. single-dose<br>Observation 6, 24 and 72 h after administration	Measurement of TBARS levels	MDA levels increased in a time-dependent manner in all brain regions, after administration of single-dose 3.5 mg OTA/kg b.w.	Sava et al., 2006a
Male Balb/C albino mice	3.5 mg/kg b.w.	i.p./ 3 days	Measurement of TBARS levels	MDA levels were significantly increased in brain tissue.	Bhat et al., 2018
Female albino Wistar rats	10 mg/kg b.w.	p.o. / 28 days	MDA detection	MDA levels were significantly increased (50%) in brain tissue.	Nogaim et al., 2020
WT zebrafish (*Danio rerio*) both sexes	1.38, 2.77, 5.53 mg/kg b.w.	i.p. / 96 h; 96 + 24 h	Measurement of TBARS levels	No changes were observed in TBARS levels after OTA administration compared to controls.	Valadas et al., 2023
Measurement of the cellular GSH status					
Male Balb/C albino mice	3.5 mg/kg b.w.	i.p. / 3 days	ABTS cation radical decolourisation assay (Re et al., 1999)	GSH status was significantly reduced after OTA administration.	Bhat et al., 2018
Female albino Wistar rats	10 mg/kg b.w.	p.o. / 28 days	GSH content assay (Jollow, 1974)	GSH levels were significantly reduced (27%) in brain tissue.	Nogaim et al., 2020
Detection of superoxide production					
Male Swiss ICR mice	0–6 mg/kg b.w.	i.p. single-dose<br>Observation 6, 24 and 72 h after administration	SOD assay (Elstner and Heupel, 1976)	Upregulation of SOD activity in all brain regions (33%), reaching a peak after 24 h and returning to control levels after 72 h, after administration 3.5 mg OTA/kg b.w.	Sava et al., 2006a
Male Balb/C albino mice	3.5 mg/kg b.w.	i.p. / 3 days	SOD assay kit (Ransod)	SOD levels were significantly decreased in OTA-treated group compared to control groups.	Bhat et al., 2018
Female albino Wistar rats	10 mg/kg b.w.	p.o. / 28 days	SOD assay (Marklund & Marklund, 1974)	SOD activity was significantly decreased in brain tissue (33%) after OTA treatment.	Nogaim et al., 2020

b.w.: body weight; GSH: glutathione; i.p.: intraperitoneal; MDA: malonaldehyde; OGG1: oxyguanosine glycosylase; OTA: Ochratoxin A; p.o.: per os; ROS: Reactive Oxygen Species; SOD: superoxide dismutase; TBARS: thiobarbituric acid reactive substances.

Supplementary Table 5 Data extraction of reviewed articles assessing **KE3: impaired proteostasis by *in vivo* techniques**

**KE3: impaired proteostasis**					
**Experimental system**	**OTA dose**	**Route/Administration period**	**Assay**	**Result**	**Reference**
Monitoring of autophagy-related molecules					
Male Balb/C albino mice	0.21, 0-5 mg/kg b.w.	p.o. by gavage / 28 days<br>Observation once a month during six months AET	WB technique	Dose-dependent decrease in LAMP-2A (20% at 0.21 mg/kg b.w. and 50% at 0.5 mg/kg b.w.) in midbrain, while no changes were observed in hsc70 protein levels.	Izco et al., 2021

AET: after the end of the treatment; b.w.: body weight; hsc70: heat shock cognate protein 70; LAMP-2A: lysosome-associated membrane protein 2A; OTA: Ochratoxin A; p.o.: *per os*; WB: Western Blot

Supplementary Table 6 Data extraction of reviewed articles assessing **KE4: degeneration of dopaminergic neurons of the nigrostriatal pathway by *in vivo* techniques**

**KE4: degeneration of dopaminergic neurons of the nigrostriatal pathway**					
**Experimental system**	**OTA dose**	**Route/Administration period**	**Assay**	**Result**	**Reference**
Dopaminergic neurons in the striatum/SNpc					
Male Balb/C albino mice	0.21, 0-5 mg/kg b.w.	p.o by gavage / 28 days<br>Observation once a month during six months AET	Immunohistochemistry (detection of TH-positive neurons)	A significant decrease of TH staining (loss of DA innervation) in the anterior and posterior striatum with both doses. A significant decrease (26%) in the number of TH+ DA neurons was observed in the midbrain after 0.21 mg OTA/kg b.w. administration.	Izco et al., 2021
Dopamine content in the striatum/SNpc					
Male Swiss ICR mice	0–6 mg/kg b.w.	i.p. single-dose /<br>Tissue sample 24 h after administration	HPLC	Dose-dependent decrease of DA striatal content (50%). ED50 of 3.2 mg/kg b.w.	Sava et al., 2006a
Male Swiss ICR mice	4, 8, 16 mg/kg b.w. (cumulative doses)	Subcutaneous continuous infusion (Alzet osotic minipumps) / 2 weeks	HPLC	A DA decline of 24% was observed in caudate/putamen, after 2 cumulative dose of 8 mg/kg b.w.	Sava et al., 2006b
Male Balb/C albino mice	3.5 mg/kg b.w.	i.p. / 3 days	RPHPLC	DA content decrease in hippocampus, striatum and whole brain tissue compared to control.	Bhat et al., 2018
Detection of Lewy Bodies (α-syn)					
Male Balb/C albino mice	0.21, 0-5 mg/kg b.w.	p.o by gavage / 28 days<br>Observation once a month during six months after the end of OTA treatment	Immunohistochemistry	p-syn aggregates in SNpc were detected (0.21 mg OTA/kg b.w.: 1.5 aggregates/section; 0.5 mg OTA/kg b.w.: 2.5 aggregates/section).	Izco *et al.*, 2021

α-syn: alpha synuclein; *AET: after the end of the treatment;* b.w.: body weight; DA: dopaminergic/dopamine; HPLC: High Performance Liquid Chromatography; i.p.: intraperitoneal; OTA: Ochratoxin A; p-syn: phosphorylated alpha-synuclein; p.o.: per os; RPHPLC: reverse-phase HPLC; SNpc: substantia nigra pars compacta; TH: tyrosine hydroxylase.

Supplementary Table 7 Data extraction of reviewed articles assessing **KE5: neuroinflammation by *in vivo* techniques**

**KE5: neuroinflammation**					
**Experimental system**	**OTA dose**	**Route/Administration period**	**Assay**	**Result**	**Reference**
Detection of astrocyte markers					
Male C57BL/6 mice	3.5 mg/kg b.w.	i.p. /1, 2, 3 or 6 doses, each dose separated by 3 days. Mice sacrificed 3 days after the last administration	Immunohistochemistry (GFAP detection)	A significant dose-dependent decrease in GFAP cell expression was observed (60% after six cumulative doses).	Mateo et al., 2022

b.w.: body weight; GFAP: Glial Fibrillary Acidic Protein; i.p.: intraperitoneal; OTA: Ochratoxin A.

Supplementary Table 8 Data extraction of reviewed articles assessing the **adverse outcome: parkinsonian motor deficits by *in vivo* techniques.**

**Adverse Outcome: parkinsonian motor deficits**					
**Experimental system**	**OTA dose**	**Route/Administration period**	**Assay**	**Result**	**Reference**
Behavioural tests					
Adult sea bass, D. lab- rax L.	0.05, 0.1, 0.15, 0.2, 0.25, 0.3, 0.35, 0.4 mg/kg b.w.	p.o. (diet; feed) / 24, 48, 72, 96h	Assessment of behavioural and swimming patters	The following behavioural changes after OTA administration were observed: sluggish movement, loss of equilibrium, rapid operculum movement as respiratory manifestations. Before death, muscular seizures occurred.	El-Sayed *et al.*, 2009
Male Balb/C albino mice	3.5 mg/kg b.w.	i.p. / 3 days	Gait analysis, spontaneous activity adhesive removal, parallel bars, pole test	<u>Gait analysis</u>: OTA treatment reduced the mean stride length measurements of forelimb and hindlimb on both 7th and 14th day of treatment.<br><u>Spontaneous activity</u>: forelimb and hindlimb steps, as well as the results of rears in cylinder and grooming time for OTA treated groups showed a lower activity on the 14th day compared to the 7th day.<br><u>Adhesive removal</u>: time taken for making contact and removing the stimulus was significantly longer for the OTA-treated groups compared to the control, even more so on the 14th day than the 7th day.<br><u>Parallel bars</u>: mice treated with OTA took significantly longer to orient themselves and to walk to one end of the pole on the 14th day compared to the 7th day, showing a decrease in motor coordination.<br><u>Pole test</u>: extrapyramidal motor dexterity was significantly affected after OTA treatment on both 7th and 14th days.	Bhat *et al.*, 2018

Supplementary Table 8 ***(Continued)*.**

Adverse Outcome: parkinsonian motor deficits					
**Experimental system**	**OTA dose**	**Route/Administration period**	**Assay**	**Result**	**Reference**
Behavioural tests					
Male Balb/C albino mice	0.21, 0.5 mg/kg b.w.	p.o. by gavage / 28 days<br>Observation once a month six months after the end of OTA treatment	Wire hang test, negative geotaxis	A significant decrease in motor performance was detected by the wire hang test and the negative geotaxis test in OTA-treated mice, 31 weeks after the end of the treatment.	Izco *et al*., 2021
WT zebrafish (Danio rerio) both sexes	1.38, 2.77, 5.53 mg/kg b.w.	i.p. / 96h; 96 + 24h	Open tank test, social interaction test	Open tank test: a significant decrease in distance, absolute turn angle and mean speed was observed with the 1.38 mg/kg dose, as well as an increase in freezing time, indicating locomotor impairment.<br>Social interaction test: OTA tested doses caused no altered social behaviour in any of the analysed parameters (distance, crossings, and interaction).	Valadas *et al*., 2023

b.w.: body weight; i.p.: intraperitoneal; OTA: Ochratoxin A; p.o.: *per o*s.

Supplementary Table 9 Data extraction of reviewed articles **non-related to any key event of the Adverse Outcome Pathway (AOP).**

Non-related to AOP KE articles					
**Experimental system**	**OTA dose**	**Route/Administration period**	**Assay**	**Result**	**Reference**
Female albino Fischer rats	0.120 mg/kg b.w.	Gastric intubation / Rats sacrificed after 10, 20 and 35 days of treatment	Determination of Ecto-Ca^{2+}/Mg^{2+}ATPase and ecto-5'Nucleotidase activity (Culić *et al*., 1990), alanine aminopeptidase activity (Jung & Scholz, 1980), γ-glutamyl transferase activity (Szasz, 1969) and N-acetyl-β-D-glucosaminidase activity	Increased activity of membrane-bound enzymes (Ecto-Ca^{2+}/Mg^{2+} ATPase, alanine aminopeptidase, ecto-5'Nucleotidase and γ-glutamyl transferase)<br>and even a larger increase in lactate dehydrogenase and N-acetyl-β-D-glucosaminidase)	Zanic-Grubisic *et al*., 1996
Male albino Wistar rats	0.289 mg/kg b.w.	Gastric intubation / 6 weeks, dose every 48h	HPLC	Tyrosine and phenylalanine levels were increased in general brain.	Belmadani *et al*., 1998 (a)
			Histological analyses	Increased number of pyknotic cells in the hippocampus.	
Male albino Wistar rats	403 ng/10 µL	Stereotaxic injection / 15 min<br>Rats sacrificed 24 h after administration	HPLC	General decrease in free amino acids levels (i.e. tyrosine) increase in the levels of phenylalanine.	Belmadani *et al*., 1998 (b)
			Determination of LDH activity (Merk kit 3399) and DNase1 activity (Kunitz, 1950)	LDH increased in mesencephalon but unaltered in the rest of the brain. DNase was increased in mesencephalon and in the rest of the brain.	
	0.289 mg/kg b.w.	p.o. by gavage / 8 days<br>Rats sacrificed 24 h after last administration	Determination of LDH activity (Merk kit 3399) and DNase1 activity (Kunitz, 1950)	LDH was increased in striatum, hippocampus, mesencephalon, and cerebellum, while DNase was increased in mesencephalon and cerebellum.	
Female SPF Wag/mbl rats	0.07, 0.34, 1.68 mg/kg b.w.	p.o. by gavage / 26-28 days	Histological analyses	Dose-dependent increase in white-matter vacuolation.	Dortant *et al*., 2001
Male Wistar rats	0.289 mg/kg b.w.	p.o. (water) / 4 weeks	WB techniques	Decreased protein expression of NMDAR subunits 2A and 2B in hippocampus.	Delibas *et al*., 2003
Male F344 (Fischer) rats	5 ppm	p.o. (diet; feed) / 35 and 51 weeks	Histological analyses	Presence of large eosinophilic bodies in the brainstem, although thought to be artifacts.	Mantle and Nolan, 2010

b.w.: body weight; HPLC: High Performance Liquid Chromatography; i.p.: intraperitoneal; LDH: lactate dehydrogenase; NMDAR: N-methyl-D-aspartate receptor; OTA: Ochratoxin A; p.o.: per os; ppm: parts per million.
